# Genus-wide analysis of *Trichoderma* antagonism toward *Pythium* and *Globisporangium* plant pathogens and the contribution of cellulases to the antagonism

**DOI:** 10.1128/aem.00681-24

**Published:** 2024-08-07

**Authors:** Siqiao Chen, Paul Daly, Wilfred Mabeche Anjago, Rong Wang, Yishen Zhao, Xian Wen, Dongmei Zhou, Sheng Deng, Xisha Lin, Josef Voglmeir, Feng Cai, Qirong Shen, Irina S. Druzhinina, Lihui Wei

**Affiliations:** 1 Key Lab of Organic-based Fertilizers of China and Jiangsu Provincial Key Lab for Solid Organic Waste Utilization, Nanjing Agricultural University, Nanjing, China; 2 Key Lab of Food Quality and Safety of Jiangsu Province—State Key Laboratory Breeding Base, Institute of Plant Protection, Jiangsu Academy of Agricultural Sciences, Nanjing, China; 3 College of Food and Bioengineering, Henan University of Science and Technology, Luoyang, China; 4 Glycomics and Glycan Bioengineering Research Center (GGBRC), College of Food Science and Technology, Nanjing Agricultural University, Nanjing, China; 5 Department of Accelerated Taxonomy, The Royal Botanic Gardens Kew, London, United Kingdom; University of Queensland, Brisbane, Australia

**Keywords:** *Trichoderma*, *Pythium*, genus-wide analysis, parasitism, exo-proteomics, *Globisporangium*

## Abstract

Parasitism is an important lifestyle in the *Trichoderma* genus but has not been studied in a genus-wide way toward *Pythium* and *Globisporangium* hosts. Our approach screened a genus-wide set of 30 *Trichoderma* species in dual culture assays with two soil-borne *Pythium* and three *Globisporangium* plant-parasitic species and used exo-proteomic analyses, with the aim to correlate *Trichoderma* antagonism with potential strategies for attacking *Pythium* and *Globisporangium*. The *Trichoderma* spp. showed a wide range of antagonism from strong to weak, but the same *Trichoderma* strain showed similar levels toward all the *Pythium* and *Globisporangium* species. The *Trichoderma* enzymes from strong (*Trichoderma asperellum*, *Trichoderma atroviride*, and *Trichoderma virens*), moderate (*Trichoderma* cf. *guizhouense* and *Trichoderma reesei*), and weak (*Trichoderma parepimyces*) antagonists were induced by the autoclaved mycelia of one of the screened *Pythium* species, *Pythium myriotylum*. The variable proportions of putative cellulases, proteases, and redox enzymes suggested diverse as well as shared strategies amongst the antagonists. There was a partial positive correlation between antagonism from microscopy and the cellulase activity induced by autoclaved *P. myriotylum* mycelia in different *Trichoderma* species. The deletion of the cellulase transcriptional activator XYR1 in *T. reesei* led to lower antagonism toward *Pythium* and *Globisporangium*. The antagonism of *Pythium* and *Globisporangium* appears to be a generic property of *Trichoderma* as most of the *Trichoderma* species were at least moderately antagonistic. While a role for cellulases in the antagonism was uncovered, cellulases did not appear to make a major contribution to *T. reesei* antagonism, and other factors are also likely contributing.

**IMPORTANCE:**

*Trichoderma* is an important genus widely distributed in nature with broad ecological impacts and applications in the biocontrol of plant diseases. The *Pythium* and *Globisporangium* genera of fungus-like water molds include many important soil-borne plant pathogens that cause various diseases. Most of the *Trichoderma* species showed at least a moderate ability to compete with or antagonize the *Pythium* and *Globisporangium* hosts, and microscopy showed examples of parasitism (a slow type of killing) and predation (a fast type of killing). Hydrolytic enzymes such as cellulases and proteases produced by *Trichoderma* likely contribute to the antagonism. A mutant deficient in cellulase activity had reduced antagonism. Interestingly, *Pythium* and *Globisporangium* species contain cellulose in their cell walls (unlike true fungi such as *Trichoderma*), and the cellulolytic ability of *Trichoderma* appears beneficial for antagonism of water molds.

## INTRODUCTION

There are only a few studies on the underlying mechanisms of the interaction between antagonistic fungi and *Pythium* or *Globisporangium* species. A summary of these studies is provided later in this Introduction, which were also reviewed recently by Chen et al. (1). The rapid expansion of agricultural use of biological control methods provides another source of studies on *Trichoderma* interactions with *Pythium* and *Globisporangium*, but these focus on applications, rather than the underlying mechanisms of the antagonism, by the use of *Trichoderma asperellum*, *Trichoderma virens*, *Trichoderma atroviride*, or *Trichoderma harzianum* to control *Pythium* or *Globisporangium*-caused tomato, Chinese-kale, sweet pepper, or tobacco diseases (2–5). The limited studies about the mechanisms underlying *Trichoderma* antagonism toward *Pythium* and *Globisporangium* species leave major gaps in understanding aspects of the microbial ecology of opportunistic parasites such as *Trichoderma* spp., as well as gaps in applications for biocontrol. Our approach was to screen well-known *Trichoderma* antagonists along with many other never-tested potential *Trichoderma* antagonists and correlate antagonism levels with potential mechanisms.

Serious threats to food security and huge economic losses are caused by soil-borne *Pythium* and *Globisporangium* plant diseases on crops worldwide (6, 7), and control of these diseases leads to irreversible environmental hazards caused by extensive fungicide use (8). Previously, the disease-causing potential of *Pythium* and *Globisporangium* (Peronosporales, Oomycota) phytopathogens has been reported, e.g., *Pythium aphanidermatum* and *Globisporangium ultimum* infecting soybean (9) and *Pythium myriotylum* infecting ginger (10). *Pythium* taxonomy has recently undergone revision, where several well-known *Pythium* plant pathogens, such as *Pythium ultimum*, have been transferred to the *Globisporangium* genus (11, 12).

The *Trichoderma* genus (Ascomycota, Hypocreales) is widely reported as among the most effective microbial antagonists and parasites (13), but their parasitic interactions with *Pythium* and *Globisporangium* species have been overlooked compared to fungal hosts. With regard to the ecological habitats, *Trichoderma* and *Pythium* as well as *Globisporangium* species could potentially interact in their shared habitats. *Pythium* and *Globisporangium* species are commonly found in soil habitats (14–16), and *Trichoderma* species tend to inhabit humus-enriched litter, rhizosphere soils, decaying wood, and habitats where their fungal hosts are abundant (17). Several studies have described the antagonism of *Trichoderma* species as shown by confrontation assays with particular phytopathogenic *Pythium* and *Globisporangium* spp. (18–20). The mechanisms underlying the antagonism and disease control are poorly understood. For example, in a recent review of *Trichoderma* parasitism, most of the examples related to fungal hosts (21).

*Trichoderma* mycoparasitism involves the production of hydrolytic enzymes such as proteases, and glucanases, as well as secondary metabolites (1, 21). Unlike fungi, the oomycete cell wall contains cellulose (≥30%) (22). The presence of cellulose in the oomycete cell wall highlights the potential importance of cellulolytic enzymes secreted from *Trichoderma* species, which are well-known industrial producers of cellulolytic enzymes (23). When *T. harzianum* antagonized *G. ultimum*, a cellulose-enriched layer was detected in *G. ultimum*, and this layer was penetrated by *T. harzianum*, supporting a role for cellulases in *Trichoderma* antagonism (24). In another study related to targeting the oomycete cell wall, the *Trichoderma reesei* signaling-related *Δgna1* deletion mutant had lower production of cell wall-degrading enzymes (CWDE) and was less antagonistic toward *G. ultimum* (25). In contrast, *T. reesei* RUT-C30 (a carbon catabolite repression-derepressed mutant with increased cellulase secretion) showed similar antagonistic levels, compared to *T. reesei* QM9978 (a cellulase induction impaired mutant without cellulase production), which suggested that the induction of cellulases was not necessary for antagonism of *G. ultimum* (26).

Other parasitism-related mechanisms that are not apparently directly related to cell wall damage have been described. Cytoplasmic coagulation was observed in the *Pythium aphanidermatum* cells after the hyphal contact with *Trichoderma* antagonists (27). With increased NADPH oxidase secretion, *T*. cf. *harzianum nox1* overexpressing mutants became more antagonistic in plate confrontation toward *G. ultimum*, compared to the control (28). Secondary metabolites secreted from *Trichoderma* species are well known for the significant inhibition of phytopathogenic fungi (29) and inhibited the growth of oomycetes such as *P. myriotylum* (30) and *P. aphanidermatum* (31).

Here, we studied the diversity of *Trichoderma*—*Pythium* and *Globisporangium* antagonistic interactions on a taxonomically wide scale. Our aim was to correlate *Trichoderma* antagonism levels with antagonism mechanisms, and in particular, to examine the relationship between antagonism levels and cellulase levels. For this purpose, we selected 30 *Trichoderma* species that represent the major infrageneric clades and investigated their antagonism toward five phytopathogenic *Pythium* or *Globisporangium* species.

## RESULTS

### Genus-wide screen shows antagonism toward *Pythium* and *Globisporangium* to be a generic property of *Trichoderma*

**Fig 1:**
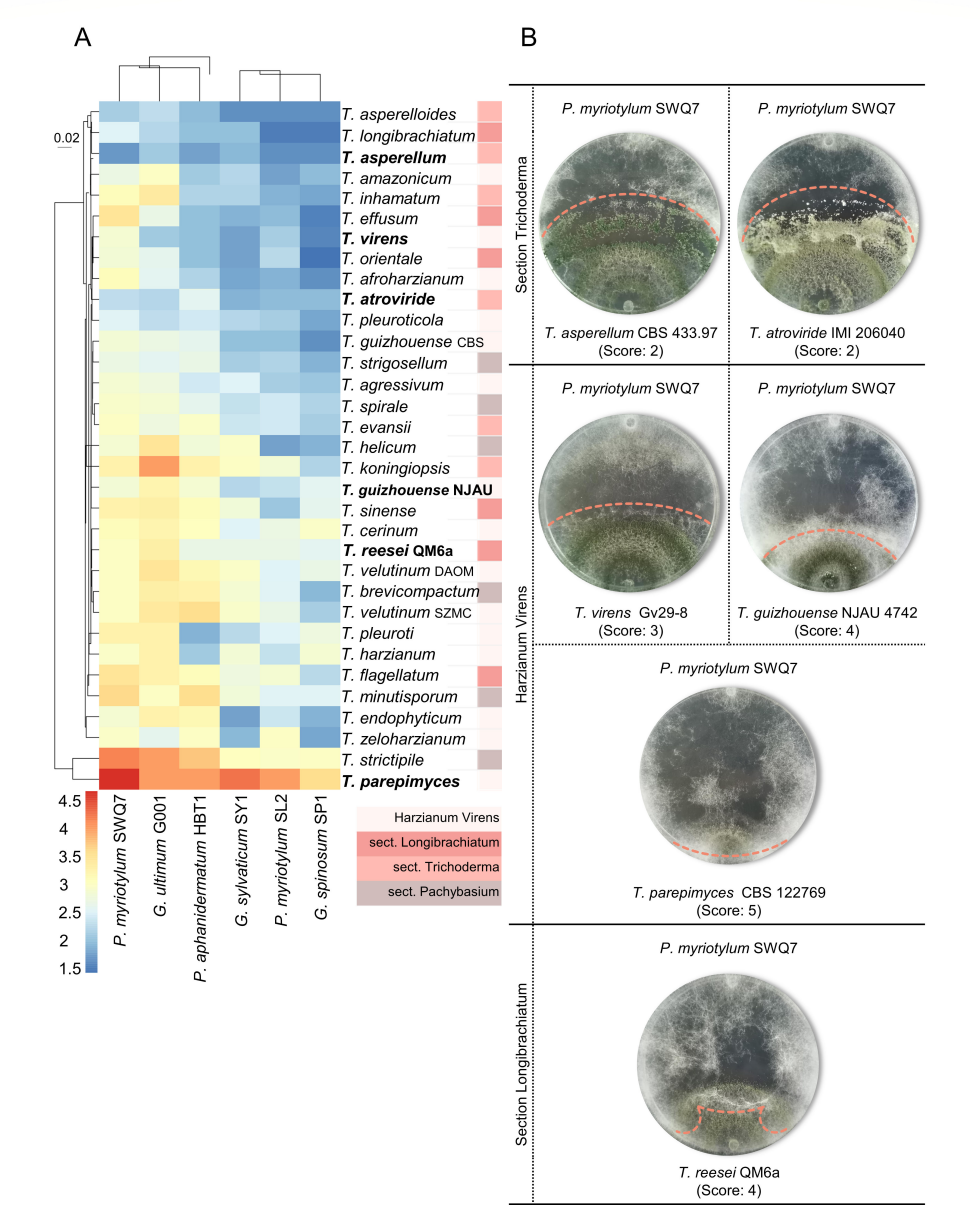
Diversity in antagonism between *Trichoderma* species and similarity in the susceptibility of the *Pythium* and *Globisporangium* isolates. (**A**) Heatmap of hierarchical clustering by the pattern of average antagonistic score evaluated for the *Trichoderma* strains in the confrontation assays, clustering by *Pythium* and *Globisporangium* in columns and *Trichoderma* in rows (cluster method: median, distance matrix method: altGower). Here, the antagonistic score is the average of the scores given to six replicates from the last five time points (every 2 d) after colonial contact. *Trichoderma* species were labeled based on the genus *Trichoderma* phylogeny with respective colored boxes. Note: “sect. Pachybasium” does not include members of the "Harzianum Virens" clade. (**B**) Representative images of *Trichoderma* spp. (bolded species in A) in the dual culture with *P. myriotylum* SWQ7 (Pm, upper) and the corresponding antagonistic scores, the coral dotted line marks the extent of overgrowth of either *P. myriotylum* or the *Trichoderma* species. The plate images were recorded 3 d after colonial contact and are representative of three plates. The data set was the average of the antagonistic scores from two independent experiments, and there was a significant positive correlation (*R*^2^ = 0.4502, *P* < 0.0001, *n* = 198) between the antagonistic scores from the first and second experiments (Fig. S1).

A selection of taxonomically diverse *Trichoderma* species (Table 1) was confronted with two isolates of the soil-borne *P. myriotylum*, one isolate of *P. aphanidermatum,* and three *Globisporangium* species (Table 2). The antagonism was scored based on the ability of one partner to overgrow the other partner. Antagonism toward the *Pythium* and *Globisporangium* species was found at varying levels across different sections of the *Trichoderma* genus (Fig. 1). Three *Trichoderma* species (*Trichoderma longibrachiatum*, and the closely related *Trichoderma asperelloides* and *T. asperellum*) had similarly strong antagonism toward all of the *Pythium* and *Globisporangium* species used in the confrontation assays. The remainder of the *Trichoderma* species had generally lower levels of antagonism with a trend whereby the antagonism tended to be stronger toward *Globisporangium sylvaticum*, *Globisporangium spinosum*, and *P. myriotylum* SL2, compared to *P. myriotylum* SWQ7, *G. ultimum*, and *P. aphanidermatum*. Only *Trichoderma parepimyces* appeared to consistently lack antagonism where it was overgrown by all of the *Pythium* and *Globisporangium* species (Fig. 1). The data set in Fig. 1 was the average of the antagonistic scores from two independent experiments, and there was a significant positive correlation (*R*^2^ = 0.5412, *P* < 0.0001, *n* = 198) between the antagonistic scores from the first and second experiments (Fig. S1). While nine of the *Trichoderma* species have been described previously from dual cultures with *Pythium* or *Globisporangium* species, to the best of our knowledge, our study is the first to culture 21 of these *Trichoderma* species in confrontation with *Pythium* or *Globisporangium* species (see Table 1 for details). Of these *Trichoderma* species that had not been studied previously, five species were strongly antagonistic to at least one of the *Pythium* or *Globisporangium* species, and seven species were moderately antagonistic toward at least one of the *Pythium* or *Globisporangium* species.

**TABLE 1:** Tested *Trichoderma* strains used in this study^*a*^

Strain ID	*Trichoderma* species	Report with *Trichoderma* species in antagonism to *Pythium* and *Globisporangium* plant-parasitic species
TUCIM 202, CBS 273.78	*Trichoderma inhamatum*	No previous report
TUCIM 254, DAOM 230007	*Trichoderma effusum*	No previous report
TUCIM 298, DAOM 230013	*Trichoderma velutinum*	No previous report
TUCIM 383, CBS 347.93, DAOM 172827	*Trichoderma strictipile*	No previous report
TUCIM 430, DAOM 230021	*Trichoderma helicum*	No previous report
TUCIM 495, DAOM 175931	*Trichoderma minutisporum*	No previous report
TUCIM 527, DAOM 230004	*Trichoderma sinense*	No previous report
TUCIM 848, CBS 115340	*Trichoderma orientale*	No previous report
TUCIM 916, CBS 226.95	*T. harzianum*	*P. myriotylum* (5, 30), *P. aphanidermatum* (32, 33)
TUCIM 917, QM6a	*T. reesei*	*G. ultimum* (25, 34)
TUCIM 1532, CBS 121216	*Trichoderma pleuroti*	No previous report
TUCIM 1540, CBS 121217	*Trichoderma pleuroticola*	No previous report
TUCIM 1680, IMI 206040	*T. atroviride*	*P. aphanidermatum* (3, 35), *G. ultimum* (36, 37)
TUCIM 1893, SZMC 27998	*T. asperelloides*	*P. aphanidermatum* (38)
TUCIM 2129, SZMC 3109	*Trichoderma aggressivum* f. sp. *europaeum*	*G. ultimum* (39)
TUCIM 2421, CBS 122769	*T. parepimyces*	No previous report
TUCIM 2924, SZMC 28001	*T. velutinum*	No previous report
TUCIM 2977, SZMC 28002	*Trichoderma cerinum*	No previous report
TUCIM 3334, SZMC 28003	*Trichoderma flagellatum*	No previous report
TUCIM 3530, Gv29-8	*T. virens*	*P. myriotylum* (40), *P. aphanidermatum* (4, 40), *G. ultimum* (41, 42)
TUCIM 3984, SZMC 28004	*Trichoderma brevicompactum*	No previous report
TUCIM 4803, T22	*T. afroharzianum*	*P. myriotylum* (30), *P. aphanidermatum* (43), *G. ultimum* and *G. sylvaticum* (44)
TUCIM 4882, IQ 11, SZMC 28005	*Trichoderma koningiopsis*	*P. aphanidermatum* (45)
TUCIM 4885, SZMC 28006	*Trichoderma evansii*	No previous report
TUCIM 4886, IQ191, SZMC 28007	*Trichoderma strigosellum*	No previous report
TUCIM 4896, MS 79, SZMC 28008	*Trichoderma spirale*	No previous report
TUCIM 4902, IB 52, SZMC 28009	*Trichoderma amazonicum*	No previous report
TUCIM 4904, T 7, SZMC 28010	*Trichoderma endophyticum*	No previous report
TUCIM 5505, SZMC 28011	*Trichoderma* sp. aff. *zeloharzianum*	No previous report
NJAU 4742	*T*. *guizhouense*	No previous report
CD22, CBS 131803	*T. guizhouense*	No previous report
H433, ATCC 18648	*T. longibrachiatum*	*P. aphanidermatum* (46), *G. ultimum* (36, 47)
CBS 433.97	*T. asperellum*	*P. myriotylum* (20, 30), *P. aphanidermatum* (2, 48), *G. ultimum* (49, 50)
Rut-C30	*T. reesei*	*G. ultimum* (26)
Rut-C30 *Δxyr1*	*T. reesei*	No previous report
Rut-C30 *xyr1* rec	*T. reesei*	No previous report

^*a*^ Strain ID is used as the culture collection code, where the strains are publicly available from these culture collections. TUCIM, TU Wien collection of industrial microorganisms; DAOM, National Mycological Herbarium, Agriculture and Agri-Food Canada; CBS, Westerdijk Fungal Biodiversity Institute; SZMC, Szeged Microbiological Collection. RUT-C30 reference strain; Δ*xyr1*, RUT-C30 *xyr1* deleted mutant; *xyr1*-rec, RUT-C30 *xyr1* recomplemented mutant.

**TABLE 2:** Tested *Pythium* and *Globisporangium* phytopathogenic isolates used in this study

Isolate ID	Collection accession	Species	*Pythium* clade	Isolated source	GenBank accession	Reference
HBT1	None	*P. aphanidermatum*	Clade A	Infected ginger rhizomes	OQ875760.1	(51)
SWQ7 SL2	CGMCC no. 21459 CGMCC no. 21956	*P. myriotylum*	Clade B	Infected ginger rhizomes	MT482756.1	(10)
SP1	None	*G. spinosum*	Clade F	Soil	OQ875759.1	(51)
SY1	None	*G. sylvaticum*	Clade F	Soil	OQ875758.1	(51)
G001	None	*G. ultimum*	Clade I	Infected ginger rhizomes	OQ875757.1	(52)

To look for trends in the antagonism related to taxonomy or ecology, the *Trichoderma* species were categorized by the taxonomic sections of the *Trichoderma* genus and three ecological categories: habitat (terrestrial or marine), lifestyle (mycoparasite, endophyte, or plant saprotroph), and distribution (cosmopolitan or limited). Subsequently, enrichment analysis was used to investigate if there was any enrichment in these terms in the strongest antagonists. There was a significant enrichment (*P* < 0.05) for cosmopolitan distribution among the *Trichoderma* species that were the strongest antagonists toward each of the *Pythium* or *Globisporangium* species (Table S3). There was also a significant enrichment (*P* < 0.05) for the *Trichoderma* sections sect. *Trichoderma* and sect. Longibrachiatum from the strongest antagonists of *P. myriotylum* SWQ7 and *G. spinosum*, respectively. Surprisingly, there was no enrichment for a mycoparasitic lifestyle in the strongest antagonists. While most of the strongest antagonists were either mycoparasites or saprotrophs, there were also species classified with these lifestyles found in similar proportions in the moderate and weak antagonists (Table S3).

For subsequent analyses to correlate the levels of antagonism with other measurements, six of the *Trichoderma* species were chosen that ranged from strong antagonists (*T. asperellum* CBS 433.97, *T. atroviride* IMI 206040, and *T. virens* Gv29-8), moderate antagonists (*T. reesei* QM6a and *T*. cf. *guizhouense* NJAU 4742), and the weakest antagonist (*T. parepimyces* CBS 122769).

### Microscopic analysis reveals *Trichoderma* parasitism of *P. myriotylum*

**Fig 2:**
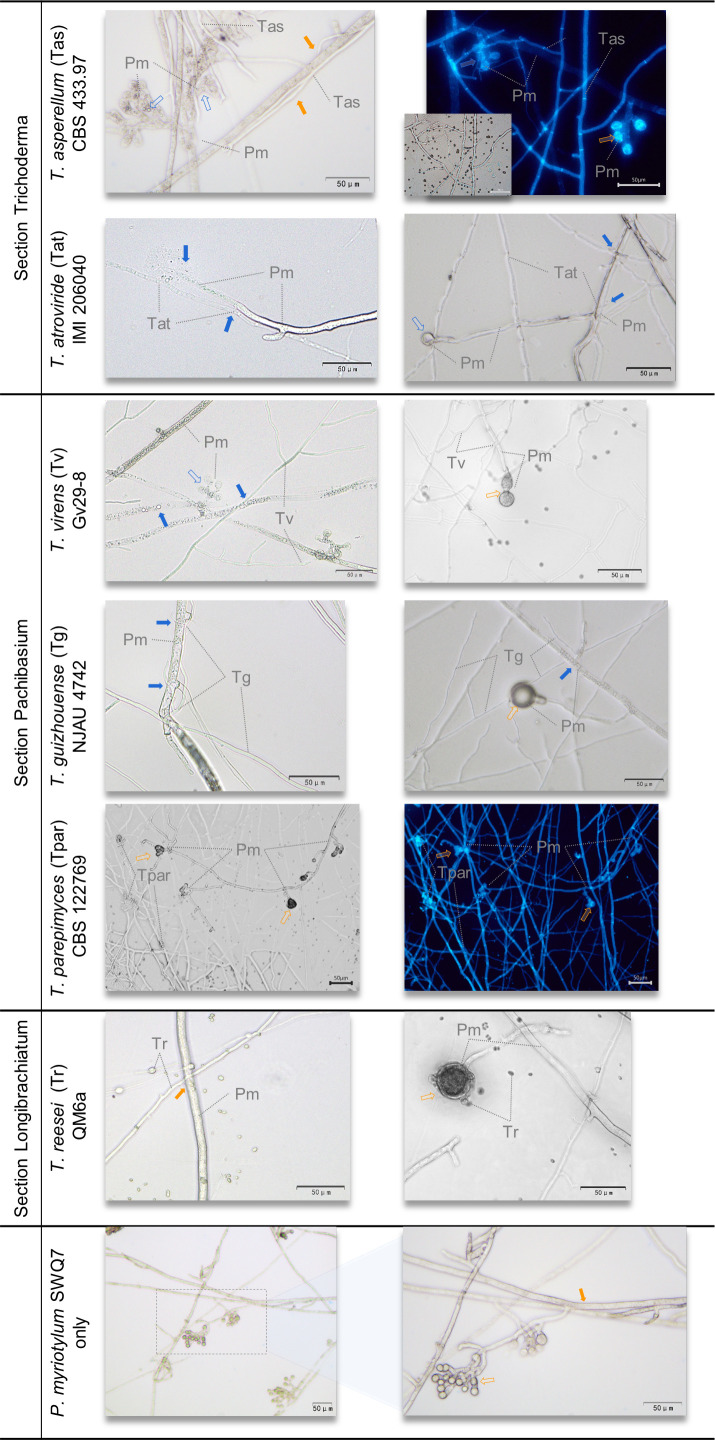
Plate confrontation and microscopic images of *Trichoderma* species (T) and *P. myriotylum* SWQ7 (Pm). The *P. myriotylum* hyphal cell wall (filled arrow) and oogonium (hollow arrow) were colored blue when damaged and orange when they appeared normal. The microscopic images were recorded 2 d after the colonial contact and are representative of five 3 cm plates. The scale bar represents 50 µm. The experiment was repeated twice with similar results each time.

Microscopy of confrontation assays between the selected *Trichoderma* strains and the selected host *P. myriotylum* SWQ7 was used to understand more about the interactions and investigate the occurrence of parasitism (Fig. 2). In the single culture of *P. myriotylum*, intact cell wall structures (orange filled arrow) and oospores (orange hollow arrow) were observed on the vigorously growing and uniform hyphal front (Fig. 2). In sharp contrast, in the dual cultures, most of the selected *Trichoderma* strains caused varying degrees of damage to the morphological structure of the *P. myriotylum* hyphae (blue filled arrow) and oospores (blue hollow arrow), as suggested from macroscopic images of the confrontation assays where there were varying degrees of uniformity and unevenness in the *P. myriotylum* colony.

*T. asperellum*, one of the strongest antagonists, quickly grew over and between the *P. myriotylum* colony to reach the other end of the plate after their hyphal contact and notably without the formation of an interlaced confrontation line. The bubbly fragments in the highly disrupted cytoplasm of *P. myriotylum* after contact with the *T. asperellum* hyphae suggested a type of cytoplasmic-targeted attack. From calcofluor white staining for polysaccharides, *T. asperellum* hyphae with many septa were distinguished from *P. myriotylum* which lacked septa (Fig. 2). The fluorescence from the cell wall of oospores surrounded by the *T. asperellum* hyphae tended to become weaker than from oospores that were not surrounded by *T. asperellum*, even though these oospores developed from the same *P. myriotylum* hypha. More than cytoplasmic damage caused by *T. asperellum* as its main antagonistic attack, *T. atroviride* and *T. virens* (two more of the strongest antagonists) caused a cell wall-targeted and a cytoplasmic-targeted attack, observed by the damaged cell wall and highly disrupted cytoplasm of *P. myriotylum*, respectively. In particular, hyphal leakage occurred at *P. myriotylum* hyphal tips near attached *T. atroviride* hyphae. Although *P. myriotylum* hyphae were damaged in the presence of the attached *T. atroviride* and *T. virens* hyphae, *P. myriotylum* was still able to form individual but thickened oospore structures. These microscopic observations of damage to *P. myriotylum* were supported by how about half of the *P. myriotylum* colony collapsed at the confrontation zone of *T. atroviride* and *T. virens* in the plate confrontation.

Fewer but thicker *P. myriotylum* oospores were also found when confronted with two moderate antagonists, *T*. cf. *guizhouense* and *T. reesei* QM6a. *T*. cf. *guizhouense* hyphae tended to wrap around *P. myriotylum* as hyphal coiling structures, causing a partial disruption of the *P. myriotylum* cell wall. In contrast, only the slightly rougher cell wall surface of *P. myriotylum* was observed after contacting *T. reesei*. Correlated to the plate confrontation images, the *T*. cf. *guizhouense* colony appeared to co-exist with the *P. myriotylum* colony around the confrontation zone and kept growing forward, whereas the *T. reesei* tended to increase sporulation and was partially overgrown by *P. myriotylum*. Finally, in the microscopic observation of *T. parepimyces* confrontations, these did not show a deleterious effect as the morphological structures of the *P. myriotylum* hyphae and the fluorescence shown from oospores were similar regardless of whether the *T. parepimyces* hyphae were close by. Similarly, in the plate confrontation, *T. parepimyces* was easily overgrown by the *Pythium* and *Globisporangium* species after colonial contact. In summary, damage to the *P. myriotylum* cell wall was observed from the following five antagonistic species suggesting a role for cell wall-degrading enzymes (the various phenomena are stated in parenthesis), *T. asperellum* (damaged cell wall implied from calcofluor white staining), *T. atroviride* (damaged hyphal tip), *T. virens* (thinner cell walls), *T*. cf. *guizhouense* (thinner cell wall), and *T. reesei* (the slightly rougher cell wall surface). Secreted *Trichoderma* proteins, such as cell wall-degrading enzymes, can be an important factor in antagonism, and the autoclaved *P. myriotylum* mycelia were used to investigate which secreted proteins were produced by these *Trichoderma* species used for the microscopic analysis that had different levels of antagonism toward the *Pythium* and *Globisporangium* hosts. Inactivated (by autoclaving) and not living *P. myriotylum* mycelia was used because it was assumed that it would not be possible to obtain exo-protein samples from weaker *Trichoderma* antagonists such as *P. parepimyces* if they were overgrown by living *P. myriotylum*. Also, hydrolytic activities such as cellulase activity from the living *P. myriotylum* mycelia would not be distinguishable from *Trichoderma* cellulases.

### Exo-proteomic analysis of *Trichoderma* shows *Pythium* mycelium can induce a diverse range of antagonism-related proteins

*P. myriotylum* mycelia were harvested, dried, and autoclaved, and preliminary experiments demonstrated that the selected *Trichoderma* species, with different levels of antagonism toward *Pythium* and *Globisporangium*, could grow on an agar medium containing the autoclaved *P. myriotylum* mycelial powder. In an analysis of the sugar composition of the acid hydrolysate of the *P. myriotylum* mycelium, glucose (94.7%) was the most abundant sugar with small amounts of mannose (2.1%), galactose (2.3%), and N-acetylglucosamine (0.9%; Table 3). The glucose is likely mainly from the cellulose, and other glucans found in oomycete cell walls (22). Liquid cultures of the *Trichoderma* species and the autoclaved *P. myriotylum* mycelia were used instead of solid cultures due to the practical ease of collecting sufficient amounts of protein for exo-proteomics analysis. PAGE gel analysis showed distinct banding patterns from the different *Trichoderma* species, suggesting that there were differences in the proteins secreted by the various species when cultured for 48 h with the same carbon source and potential inducer of the secreted proteins (Fig. S2). Based on the comparisons of the banding patterns between the species, four species (*T. atroviride*, *T*. cf. *guizhouense*, *T. reesei* QM6a, and *T. virens*) were strongly induced in liquid culture to secrete a range of proteins as shown by the strong protein bands on the PAGE gel, while the other two species (*T. asperellum* and *T. parepimyces*) had much weaker protein bands.

**TABLE 3:** Sugar compositional analysis of the extracted *P. myriotylum* mycelial powder^*a*^

Monosaccharide	Glucose	Galactose	Mannose	N-acetyl glucosamine	Xylose	Arabinose	Rhamnose
Mol values (%)	94.85	2.28	2.1	0.76	ND^*b*^	ND	ND
SE (%)	0.17	0.06	0.13	0.05	ND	ND	ND

^*a*^ Errors are the SE from the sugar quantification from three technical replicate hydrolysates.

^*b*^ ND, not detected.

The exo-proteomic analysis quantified between 232 and 628 proteins from *T. asperellum* (273), *T. atroviride* (310), *T*. cf. *guizhouense* (232), *T. parepimyces* (628), *T. reesei* QM6a (284), and *T. virens* (278). There were between 79 and 150 of the proteins that were predicted to be extracellular with relative abundance between 61.6% and 91.1% which indicated relatively healthy mycelia from *T. asperellum* (133 proteins and 61.6%), *T. atroviride* (133 proteins and 84.0%), *T*. cf. *guizhouense* (150 proteins and 83.2%), *T. parepimyces* (124 proteins and 67.6%), *T. reesei* QM6a (116 proteins and 72.0%), and *T. virens* (104 proteins and 91.1%).

**Fig 3:**
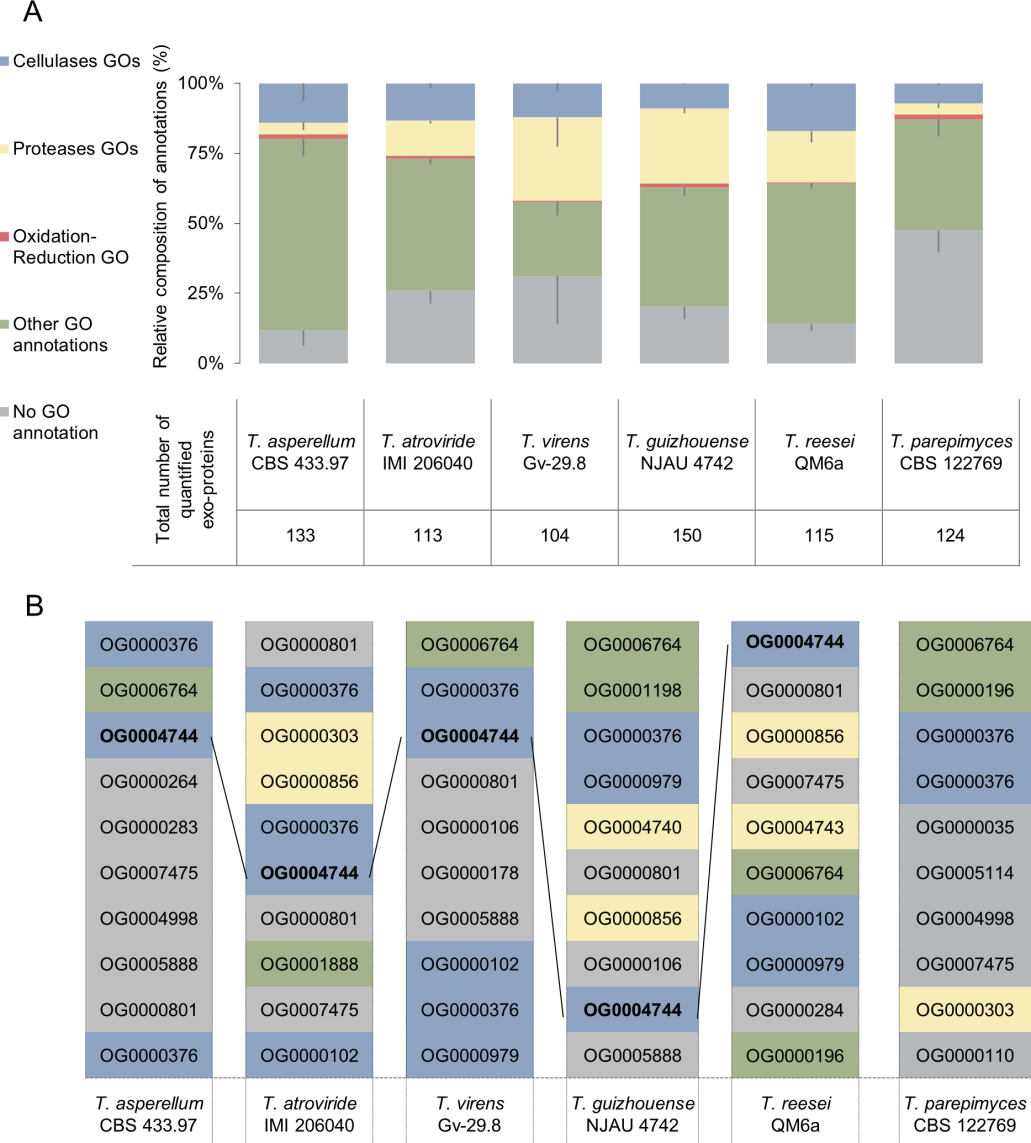
Potential antagonistic factors revealed in proteomic and orthogroup analysis of *Trichoderma* exo-proteins. Proteins with “extracellular only” annotation were included in the analysis. (**A**) Diversity in the proportion of major antagonism-related GO functional categories in the exo-proteins secreted by the six *Trichoderma* species as measured by the percentage of the label-free quantification intensity values. Cellulases GOs (in blue): GO:0030248(MF:cellulose binding), GO:0030245(BP:cellulose catabolic process), and GO:0008810(MF:cellulase activity). Proteases GOs (in yellow): GO:0008233(MF: peptidase activity) and GO:0006508(BP: proteolysis). Oxidation-reduction GO (in red): GO:0055114(BP: oxidation-reduction process). The error bars represent SE (*n* = 3). (**B**) Diversity in the regulation of orthologous proteins from core orthogroups (where at least one orthologous protein is present in all six of the *Trichoderma* genomes). The exo-proteins are ranked by abundance for each species and labeled by orthogroup to demonstrate changes in the relative abundance of orthologous exo-proteins in each species. The major GO functional categories are color-coded in (**A**) and (**B**). The black lines in (**B**) show the abundance trend of detected cellobiohydrolases from a conserved orthogroup.

Gene ontology (GO) terms were used to annotate the *Trichoderma* proteins to facilitate a cross-species comparison of the potential antagonistic strategies used by *Trichoderma*. Several broad-based GO terms for proteolysis, oxidation-reduction processes, and cellulases were used to indicate potential antagonistic strategies. *Trichoderma* proteases can function to hydrolyze structural proteins in the cell wall of *P. myriotylum*, such as cell membranes, and other *P. myriotylum* proteins. Remarkably, ~30% were annotated for proteolysis from exo-proteins of *T. virens* and *T*. cf. *guizhouense* and lower proportions from the other *Trichoderma* species with *T. parepimyces* having the lowest proportion of proteolysis-related proteins of all the species at 3.9% (Fig. 3A; Table S4). *Trichoderma* cellulases can function in the hydrolysis of the cellulose component of the *P. myriotylum* cell wall. The *Trichoderma* cellulase-related proteins were proportionally most abundant from *T. reesei* with 17% and least abundant from *T. parepimyces* with 7.1% abundance (Fig. 3A; Table S4).

Alongside the comparison of GO annotations, orthologous protein comparisons were used to understand whether orthologous proteins were produced at the same or different levels across the *Trichoderma* species. First, the ortholog analysis of all the proteins predicted in the genomes from the selected *Trichoderma* species identified between 7,682 and 8,311 core proteins from *T. asperellum* (8,036), *T. atroviride* (8,063), *T*. cf. *guizhouense* (8,117), *T. parepimyces* (8,009), *T. reesei* QM6a (7,682), and *T. virens* (8,311) that were found across a total of 7,268 core protein orthogroups. There were 3,957 dispensable protein orthogroups and 5,229 unique protein orthogroups in a total of 16,454 orthogroups (Table S1). Note that the terms core (present in all genomes), dispensible (present in at least two genomes), and unique (present in only one genome) refer to the genome content and not the presence or absence in the detected exo-proteins from the cultures. There were 51 core genome orthogroups where the orthologous protein was indeed detected from the culture filtrate from all six *Trichoderma* species, and notably, most of these core orthogroups were predicted to contain extracellular proteins.

There were 24 core genome orthogroups ranked in the lists of the top 10 most abundant core genome orthogroup proteins across the culture filtrates of the six species, and none of these 24 core genome orthogroups were found in the top 10 lists of all six *Trichoderma* species (Fig. 3). This implies differences in the regulation of gene expression or post-transcriptional regulation of orthologous *Trichoderma* proteins when cultured with the autoclaved *P. myriotylum* mycelia. These top 10 ranked core orthogroup proteins represented a majority in the total abundance of the extracellular proteins from all six *Trichoderma* species (ranging from 64.3% to 84.3%). The following particular proportions of the abundance of the top 10 ranked core orthogroup proteins were found in each species: *T. asperellum* (64.3%), *T. atroviride* (66.5%), *T*. cf. *guizhouense* (66.3%), *T. parepimyces* (71.7%), *T. reesei* QM6a (74.5%), and *T. virens* (84.3%). Regarding cellulases, the β-glucosidases from OG0000376 orthogroup appear to be one of the most conserved abundantly produced proteins. OG0000376 orthogroup members (which contain proteins annotated as GH3 putative β-glucosidases, and the GO term for cellulose catabolic process) were detected in the exo-proteins from all of the selected species, except for *T. reesei* QM6a. The OG0004744 members (which contain proteins annotated as GH7 cellulase proteins) were detected in the secreted proteins from all of the selected species, except for *T. parepimyces*.

For correlating potentially antagonistic secreted proteins with the confrontation assay, it was noteworthy that GH7 (CAZy Glycoside Hydrolase Family 7, IPR001722) cellulases were present in the exo-proteins from all of the *Trichoderma* species except for *T. parepimyces* (Table S4) which was the weakest antagonist in the confrontation assays. Interestingly, according to the Orthofinder analysis of the *Trichoderma* genomes (Table S1), the *T. parepimyces* genome has two GH7-containing orthogroups, the core orthogroup OG0004744 (containing Tripare1_369787) and the dispensable orthogroup OG0008108 (containing Tripare1 361284). Neither of these *T. parepimyces* proteins was detected in the exo-proteomics. This implies differences in the regulation of gene expression or post-transcriptional regulation of GH7 cellobiohydrolases when cultured with the autoclaved *P. myriotylum* mycelia.

The variable levels of cellulases between stronger and weaker antagonists coupled with the presence of cellulose in the *P. myriotylum* cell wall suggested that cellulase activity could be an important factor in the *Trichoderma* antagonism of *Pythium* and *Globisporangium* hosts. The shake-flask cultures have two key limitations when trying to extrapolate to plate-based confrontations (the cultures were in liquid not plate-based, and there was no living host). To overcome these, the cellulase activity in plate-based cultures was measured using single cultures, and a *Trichoderma* mutant with reduced cellulase activity was used in confrontation assays with *Pythium* and *Globisporangium* hosts.

### Partial correlation of cellulase activity in plate cultures with the level of antagonism

**Fig 4:**
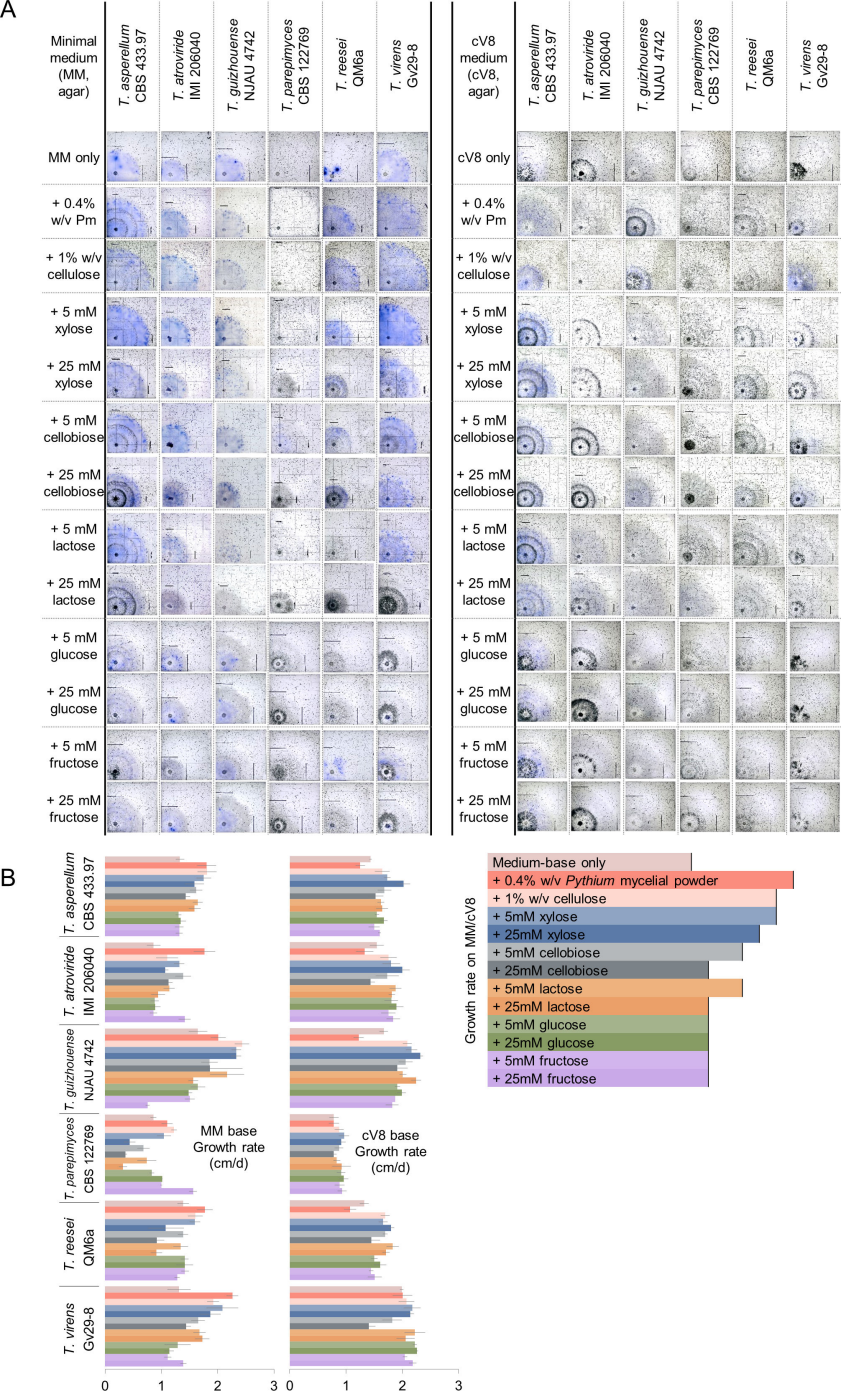
The AZCL cellulase assays of *Trichoderma* species on the minimal medium and cV8 medium containing potential cellulase inducers: 0.4% (wt/vol) *P*. *myriotylum* mycelial powder (Pm), 1% (wt/vol) cellulose, 5 mM, and 25 mM of cellobiose, D-xylose, lactose, or the non-inducing D-glucose, and D-fructose. (**A**) Plate culture images of AZCL cellulase assays are representative of three plates. The black lines indicate the location of the hyphal front of the colony. (**B**) The growth rate of the *Trichoderma* colony in AZCL cellulase assays (represented by the bar chart, where the error bars represent SEs from three replicate plates).

AZCL-cellulose is a blue-dyed chromogenic cellulose polymer, whereby the hydrolysis of the cellulose is detected by the intensity and spread of the blue dye in agar plates. The various levels of the detectable degradation of this dyed polymer showed the endo-cellulase activity induced in the selected *Trichoderma* strains growing on the various media (Fig. 4). On the cV8 medium alone, which was also used in the confrontation assays, cellulase activity was only detected from *T. asperellum*. To make the conditions for testing for cellulase activity more similar to that in the confrontation assays, autoclaved *P. myriotylum* mycelial powder was added to the cV8 medium. Endo-cellulase activity was detected from *T. asperellum*, *T. virens*, and *T*. cf. *guizhouense*. However, no endo-cellulase activity was detected from *T. atroviride*, *T. parepimyces*, and *T. reesei* (Fig. 4A). While the growth rate of *T. parepimyces* was slower, the growth rates of *T. atroviride* and *T. reesei* were comparable to the other *Trichoderma* species indicating that the lack of detectable endo-cellulase activity was not due to lower growth rates (Fig. 4B). The endo-cellulase activity induced by the addition of *P. myriotylum* mycelial powder to cV8 medium partly positively correlated with the damage to the *P. myriotylum* cell wall in the microscopy. For example, the following lists the species and a relative scoring of the cellulase activity and cell wall damage from the microscopy in parenthesis: *T. asperellum* (++, damaged cell wall), *T. atroviride* (—, damaged hyphal tip), *T*. cf. *guizhouense* (+++, thinner cell wall), *T. reesei* QM6a (—, slightly rougher cell wall surface), *T. parepimyces* (—, no damage), and *T. virens* (+++, thinner cell walls).

It was thought that the reason for lack of detectable cellulase activity could be because cV8 medium and *P. myriotylum* mycelial powder were not good cellulase inducers, and instead, the cV8 medium was supplemented with other potential inducers of cellulase activity (purified cellulose, cellobiose, lactose, and D-xylose). Still, the trend was not noticeably different, whereby still little or no cellulase activity was detected from *T. atroviride*, *T. parepimyces*, and *T. reesei* QM6a. The lack of detectable cellulase activity from the *T. reesei* QM6a strain was surprising, and it was thought that the presence of potential carbon catabolite repressors of cellulase activity in the cV8 medium may have been responsible. One of the most abundant sugars in V8 is D-glucose which has been measured at 0.7% (wt/vol) previously by Kent et al. (53) and can be repressive of cellulase gene expression in *Trichoderma* species. When the cV8 medium was replaced by a minimal medium containing various cellulase inducers as the carbon source, cellulase activity was detected from the *T. reesei* QM6a strain supporting the occurrence of repressive effects from the cV8 medium. Cellulase activity was detected from most of the species on all of the cellulase inducers on minimal medium except for *T. parepimyces* which only had visibly detectable levels of cellulase activity when 5 mM cellobiose was used as the inducer (Fig. 4A). *T. parepimyces* could grow on these potential cellulase inducers, albeit the growth was slow, indicating that the lack of detectable endo-cellulase activity was not due to a lack of *T. parepimyces* growth (Fig. 4B).

### Antagonism toward *Pythium* and *Globisporangium* reduced by using a cellulase deficient *T. reesei* Δ*xyr1* mutant

**Fig 5:**
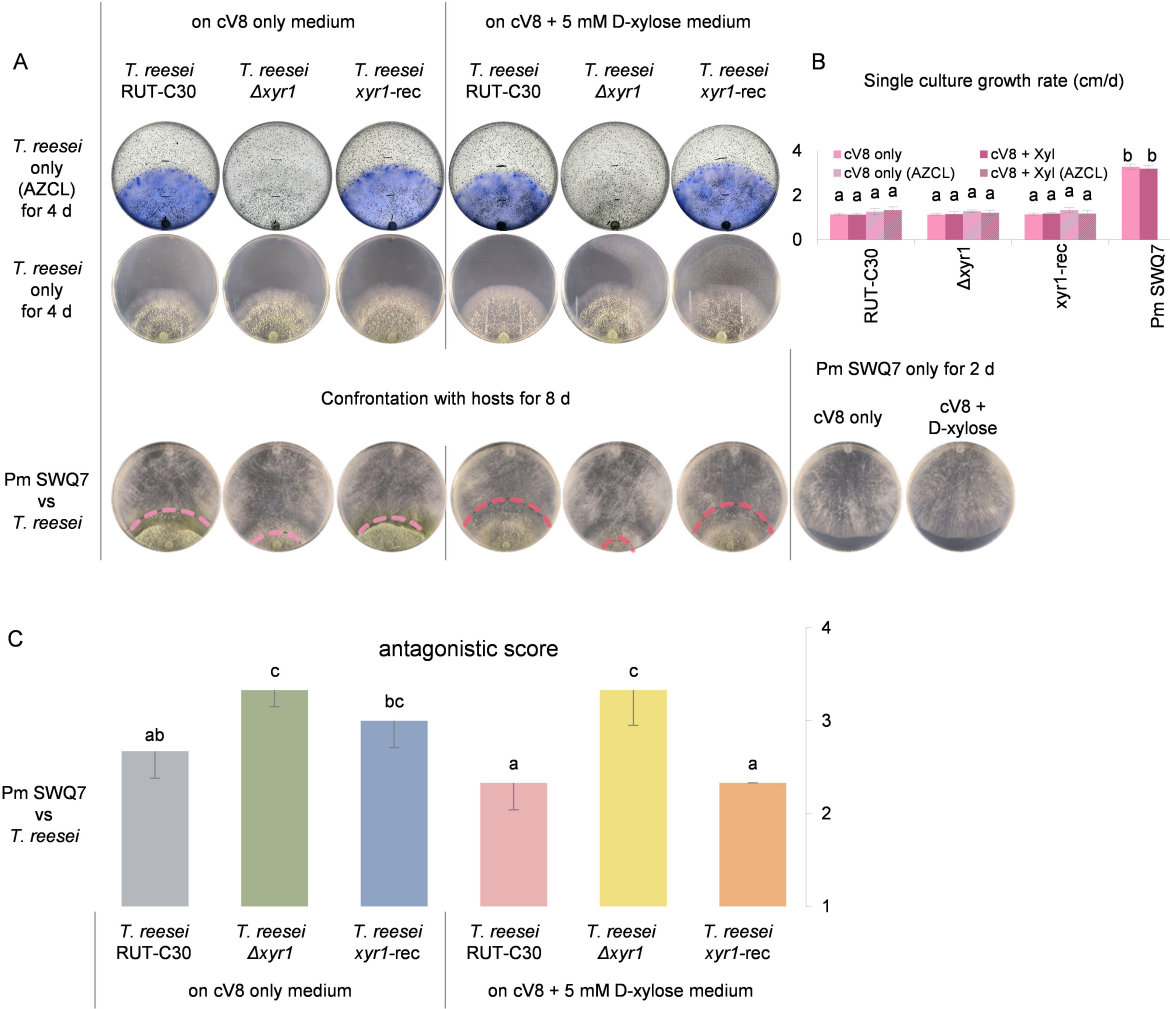
Potential contribution of cellulase in the antagonism of *P. myriotylum* (Pm). RUT-C30, reference strain; Δ*xyr1*, RUT-C30 *xyr1* deleted mutant; *xyr1*-rec, RUT-C30 *xyr1* recomplemented mutant. (**A**) Plate culture images of *T. reesei* RUT-C30 mutants and *P. myriotylum* SWQ7 on cV8 and its 5 mM D-xylose supplemented medium. Dual culture images were recorded 3 d after the colonial contact, alongside an antagonistic score represented by the bar chart in part (**C**). The confrontation front was labeled with a dotted line. On the AZCL plates, the short, continuous black lines located furthest away from the inoculation point indicate the location of the hyphal front of the colony when the photo was taken. (**B**) Single culture growth rate (cm/d) of the tested *T. reesei* and *P. myriotylum* strains, and error bars represent SE. (**C**) Bar chart of the average antagonistic score evaluated for the *T. reesei* RUT-C30 mutants in the confrontation assays from the last five time points (every 2 d) after colonial contact, and error bars represent SE. These experiments were repeated two times with similar results each time. Confrontation assays between *T. reesei* RUT-C30 mutants and five other *Pythium* and *Globisporangium* isolates are shown in Fig. S3.

The analysis of the contribution of cellulases to *Trichoderma* antagonism is complicated by how the *Pythium* and *Globisporangium* hosts also produce cellulases, and usage of the cellulase inhibitor would probably reduce the fitness of the *Pythium* and *Globisporangium* hosts. Instead, a mutant in a transcriptional activator XYR1 (xylanase regulator 1) of *Trichoderma* cellulase gene expression was used. In a previous study, the *xyr1* gene was deleted in the *T. reesei* RUT-C30 strain by Ma et al. (54), and we used this deletion mutant to investigate if reduced cellulase gene expression could reduce the *T. reesei* antagonism toward *Pythium* and *Globisporangium* species. The use of the *T. reesei* RUT-C30 background was also critical for this investigation because RUT-C30 is carbon catabolite repression-derepressed. The repression of the cellulase activity on the cV8 medium was alleviated as shown in Fig. 5. Also, in contrast to the *Trichoderma* strains described in the previous section and Fig. 4, the RUT-C30 parental strain showed much stronger cellulase activity on the cV8 medium.

In initial tests, the AZCL-cellulose activity assays showed that the endo-cellulase activity was undetectable in the Δ*xyr1* mutant compared to the controls and without any significant reduction in the growth rate of the mutant (Fig. 5). In confrontation assays between *P. myriotylum* SWQ7 and the *T. reesei* RUT-C30 Δ*xyr1* mutant and controls, there was reduced levels of antagonism from the Δ*xyr1* mutant toward *P. myriotylum* SWQ7, as measured by reduced plate coverage of the Δ*xyr1* mutant and increased coverage of *P. myriotylum* (Fig. 5). However, the detected but limited delay of antagonism from the Δ*xyr1* mutant compared to controls did not convincingly capture the contribution of *T. reesei* cellulases. Instead, the use of increased cellulase activity and potentially stronger antagonistic ability was attempted by supplementing the medium used for dual cultures with D-xylose which is one of the major cellulase inducers in *T. reesei* (55). When induced with D-xylose in the cV8 medium in the single culture, there was a slight increase in endo-cellulase activity in the control strains, while the cellulase activity was still undetectable in the Δ*xyr1* mutant (Fig. 5). On these cV8 plates supplemented with D-xylose, the delay in antagonistic ability became more obvious between the Δ*xyr1* mutant compared to the controls in the confrontation assay toward *P. myriotylum* SWQ7. The Δ*xyr1* mutant was overgrown similarly in dual cultures with or without the D-xylose supplementation, while both of the control strains showed increased antagonism in confrontation assays as shown by the bigger difference between the Δ*xyr1* mutant and control on the medium supplemented with D-xylose (Fig. 5). Of note, the *T. reesei* Δ*xyr1* mutant and controls did not show significant differences in their growth rate on cV8 supplemented with xylose.

The approach used above was also tested with another *P. myriotylum* isolate SL2, and the other *Pythium* and *Globisporangium* species used in the confrontation screen that started our study, *P. aphanidermatum*, *G. spinosum*, *G. sylvaticum*, and *G. ultimum* (Fig. S3). There was no clear *Pythium* or *Globisporangium* species-dependent effect of the Δ*xyr1* mutant on the antagonism because the Δ*xyr1* mutant had similarly reduced levels of antagonism toward all the *Pythium* and *Globisporangium* species (Fig. S3). Similar to the confrontation assays with *P. myriotylum* SWQ7 strain on the cV8 plates supplemented with D-xylose, the delay in antagonistic ability became more obvious between the Δ*xyr1* mutant compared to the controls in the confrontation assay toward all of the *Pythium* and *Globisporangium* hosts (Fig. S3).

### *T. asperellum* can control Pythium soft rot of ginger

As well as investigating the underlying mechanisms of the *Trichoderma* antagonism toward *Pythium* and *Globisporangium* species, it was of interest whether the genus-wide screen could identify *Trichoderma* strains that could control a disease caused by one of the *Pythium* species. *P. myriotylum* can cause soft-rot disease in ginger, and the ability of one of the strongest antagonists, *T. asperellum*, to control *P. myriotylum-*caused soft-rot disease in ginger was investigated (Fig. S4). The ginger plants were first inoculated with a *Trichoderma* spore suspension followed by inoculation with the *P. myriotylum* SWQ7 isolate. The plants inoculated with only *P. myriotylum* died, demonstrating the high virulence of the *P. myriotylum* SWQ7 isolate (Fig. S4). The use of the higher spore concentration inoculum of *T. asperellum* led to a disease protection rate of ~50% compared to ~25% using the lower spore concentration inoculum (Fig. S4). The presence of the inoculated microbes at the end of the pot-trial was confirmed by re-isolation. *P. myriotylum* was re-isolated from the dead seedlings infected by *P. myriotylum*, and *T. asperellum* was re-isolated from the disease-protected seedlings that were inoculated with both *P. myriotylum* and *T. asperellum*, and the identification was confirmed by sequencing of the ITS region.

## DISCUSSION

Here, we showed that antagonism toward *Pythium* and *Globisporangium* appears to be a generic property of *Trichoderma*, various antagonism-related proteins are induced by *P. myriotylum* mycelium, and cellulases are a contributing factor to the antagonism of *Pythium* and *Globisporangium* species.

The ancestral lifestyle of *Trichoderma* is considered to be mycoparasitism (56), and later, *Trichoderma* evolved to degrade plant cell walls via horizontal gene transfer of plant cell wall-degrading enzymes including cellulases (57). The antagonism toward *Pythium* and *Globisporangium* appears to be a generic property of *Trichoderma* because most of the *Trichoderma* species were at least moderately antagonistic toward *Pythium* and *Globisporangium*. Also, there was limited variability in the antagonism toward different *Pythium* or *Globisporangium* species, suggesting that it is part of the well-known environmental opportunism of *Trichoderma* species rather than any specific *Trichoderma-Pythium* and *Globisporangium* interaction. The evolution of *Trichoderma* to degrade plant cell walls could have also facilitated parasitism of cellulose-containing *Pythium* and *Globisporangium* species becoming a generic property of *Trichoderma*.

Although the antagonism toward *Pythium* and *Globisporangium* appears to be a generic property of *Trichoderma*, there were some suggestions from the enrichment analysis of lifestyle or *Trichoderma* section-related enrichments in the strength of the antagonism. From the enrichment analysis of the strongest *Trichoderma* antagonists, there was significant enrichment (*P* < 0.05) for cosmopolitan distribution among the *Trichoderma* species that were the strongest antagonists toward each of the *Pythium* or *Globisporangium* species (Table S3). Most of the stronger antagonists, such as *T. asperellum*, *T. asperelloides*, *T. longibrachiatum*, *T. virens*, *T. atroviride*, *T. pleuroticola*, and *T. orientale*, have a worldwide distribution (57–60). It is perhaps more likely for the *Trichoderma* species with cosmopolitan distribution to interact with the *Pythium* and *Globisporangium* species in nature sufficiently to evolve effective antagonistic strategies. Soil-like habitats are common to *Pythium* and *Globisporangium* species (14), which is similar to the humus-enriched litter or decaying wood where cosmopolitan *Trichoderma* species tend to inhabit (17). The sect. *Trichoderma* was significantly enriched in the strongest antagonists of *P. myriotylum* SWQ7, and the sect. *Longibrachiatum* was significantly enriched in the strongest antagonists of *G. spinosum*, suggesting there might be some taxonomically related selection in the strength of antagonism toward particular *Pythium* or *Globisporangium* species. It is important to note that these types of enrichment analyses depend on the reliability of the distribution, lifestyle, and habitat categorizations of the species and are subject to change as more information about lesser-studied species emerges. The raw data from the scoring of the antagonism are available to facilitate future analyses (Table S2).

Interestingly, several of the strongest antagonists (*T. asperellum*, *T. asperelloides*, and *T. longibrachiatum*) have a plant saprotrophic lifestyle as well as a lifestyle related to antagonism (61). This suggested that the plant saprotrophic lifestyle might contribute to their strong antagonism to *Pythium* and *Globisporangium* species. Unlike the cell wall of true fungi, both oomycetes and plants have cellulose in their cell walls (22, 62). It might provide an extra advantage for strong antagonists if they also have a plant saprotrophic lifestyle, which facilitates degradation of the cellulose-enriched *Pythium* and *Globisporangium* cell wall. There was support for a role for cellulases from the partial positive correlation between the levels of endo-cellulase activity on cV8 medium supplemented with the autoclaved *P. myriotylum* mycelia and the cell wall damage observed in the microscopy. However, it was found that the moderate and weak antagonists did not benefit a lot from their plant saprotrophic lifestyles, which implied that other factors along with cellulose degradation are important for the strong antagonists of *Pythium* and *Globisporangium* species. Previous studies provide support for the contribution of secreted *Trichoderma* cell wall-degrading enzymes. The *T. reesei* Δ*gna1* deletion mutant with lower production of cell wall-degrading enzymes was less antagonistic toward *G. ultimum* (25). Extracellular hydrolytic assays for the potential *Pythium* cell wall-targeted enzyme, cellulases, β−1,3-glucanases, and proteases, were correlated positively with the antagonistic levels of 20 *T. harzianum* isolates toward *P. aphanidermatum* (18).

*T. asperellum*, which was ranked as one of the strongest antagonists toward all the *Pythium* and *Globisporangium* hosts, has been reported previously as an antagonist toward *P. myriotylum* (20, 30), *P. aphanidermatum* (2, 48, 63), and *G. ultimum* (49, 50, 64). The cell damage from the microscopy analysis suggests that *T. asperellum* parasitism may be contributing to the antagonism of *P. myriotylum*. The weaker oospore polysaccharide fluorescence when surrounded by *T. asperellum* hyphae suggests cell wall damage, and there were bubbly fragments within the *P. myriotylum* hyphae (Fig. 2). In the related oomycete *Phytophthora capsici*, its cytoplasm was also damaged showing bubbly fragments upon contact with *T. asperellum* (65). Also, *T. asperellum* controlled *P. myriotylum*-caused ginger soft rot disease (Fig. S4). Previously, *T. asperellum* strongly controlled the *P. myriotylum*-caused plant diseases in cocoyam root rot (20). Other strong *Pythium* and *Globisporangium* antagonists (e.g., *T. asperelloides*, *T. pleuroticola*, *T. amazonicum*, *T. inhamatum*, *T. effusum*, and *T. orientale*) warrant future testing for their ability to control *Pythium*- and *Globisporangium*-caused diseases.

Some aspects of the interaction between *P. myriotylum* and *T. atroviride* or *T. virens* in our study were similar to previous studies in which *G. ultimum* was the host. In our study, *T. atroviride* hyphae tended to extend alongside the *P. myriotylum* hyphae, and the *P. myriotylum* hyphal tip was damaged, and at the macroscopic level, *T. atroviride* sporulated on the collapsed *P. myriotylum* colony. This was similar to how *T. atroviride* hyphae extended alongside *G. ultimum* where spores also attached to the *G. ultimum* hyphae (66). In our study, there was cytoplasmic coagulation within the *P. myriotylum* hyphae along with thinner cell walls. Previously, *G. ultimum* cytoplasmic coagulation and disintegration were found when growing close to *T. virens* hyphae (67). The thinning of the cell wall of *P. myriotylum* hyphae might be caused by the cell wall-targeted attack of *T. virens*, and this correlated with the strong *T. virens* endo-cellulase activity induced by the *P. myriotylum* mycelial powder (Fig. 4).

*T. reesei* QM6a showed a below-the-average moderate antagonistic level where *T. reesei* QM6a took 4–6 d to reach a deadlock when confronted with the *Pythium* and *Globisporangium* species. Previously, *T. reesei* TU-6 moderately inhibited the growth of *G. ultimum* and formed a deadlock (34). In contrast to *T*. cf. *guizhouense*, *T. reesei* showed less interaction with the overgrown *P. myriotylum* aerial mycelia but strongly increased sporulation. Perhaps the sporulation is part of a tolerance strategy against biotic stress (68). *T. reesei* caused *P. myriotylum* mycelia to collapse only at the colonial front, and *T. reesei* hyphae caused no obvious damage to *P. myriotylum* hyphae except for the slightly rougher cell wall, where numerous spores spread unusually upon hyphal contact. In several previous studies, SEM images showed a contribution from CWDEs, whereby *T. reesei* TU-6 caused holes characteristic of CWDE activity in the *G. ultimum* cell wall, and *T. reesei gna1*QL mutants with the higher secretion of CWDEs produced more holes (25).

As expected, the *T. reesei* RUT-C30 *xyr1* deletion mutant (Δ*xyr1*) showed undetectable endo-cellulase activity supporting how XYR1 is a major transcriptional activator of *Trichoderma* cellulases (54, 69). The RUT-C30 Δ*xyr1* mutant showed a delayed antagonism to all the *Pythium* and *Globisporangium* species compared to its controls, and a larger delay in antagonistic levels was found when xylose was added as a cellulase inducer. These cellulase-related observations using the *T. reesei* Δ*xyr1* mutant highlighted the contribution of cellulases to the cell wall-targeted strategy. The delayed antagonism of the *T. reesei* RUT-C30 Δ*xyr1* mutant is in contrast to a previous study in another *Trichoderma* species. A *T. atroviride* Δ*xyr1* mutant appeared as the better antagonist as measured by its slightly quicker growth and increased sporulation in confrontation with *G. ultimum* (70). However, it may also be possible to speculate from their results that the degradative ability of the *T. atroviride* Δ*xyr1* mutant may have been lower as there appeared to be more *G. ultimum* mycelia remaining in the confrontation zone (70). Another study using a *T. reesei* mutant (deficient in cellulase induction) suggested that induction of cellulases was not necessary for the *T. reesei* RUT-C30 antagonism of *G. ultimum* (26). The *T. reesei* RUT-C30 strain did not show better antagonism toward *G. ultimum* than the *T. reesei* QM9978 mutant, which was impaired in cellulase induction (26). Meanwhile, the data related to the *T. reesei* Δ*xyr1* mutant in our study suggests that there could be a contribution of cellulases, particularly where xylose is used as a cellulase inducer (Fig. 5). Since the tested *xyr1* mutant in our study was in a moderate antagonist, for further investigating the contribution of cellulases to antagonism, it would be of interest to delete *xyr1* in strong *Trichoderma* antagonists.

As the exo-proteomic analysis and orthogroup analysis stated in the result section, there was a positive correlation to the weakest antagonism of *T. parepimyces* in plate confrontation and the absence of GH7 putative cellobiohydrolases which appeared to be one of the main cellulases present that could be involved in degrading the cellulose polymer found in the *Pythium* and *Globisporangium* cell wall. The absence of detected *T. parepimyces* GH7 putative cellobiohydrolases might be caused by the different signaling or secretion pathways, compared to the other selected species because its genome encodes for these proteins. Previously, it has been shown in Aspergilli that protein production of an orthologous protein can vary between different species when the same inducer or carbon source is used (71). However, it should be noted that the trend in GH7 cellulase production in the liquid cultures with minimal medium and autoclaved *P. myriotylum* mycelia may not be the same as the cV8 plate cultures where there was a biotic interaction with *Pythium* or *Globisporangium* species. Apart from cellulases, other factors are likely contributing such as the cytoplasmic damage that was observed in the antagonism toward *P. myriotylum*, which can be listed in the strong and moderate antagonists (shown with various cytoplasmic phenomena in parenthesis), *T. asperellum* (highly disrupted cytoplasm with bubbly fragments), *T. atroviride* (slightly cytoplasmic coagulation), *T. virens* (strongly cytoplasmic coagulation), and *T*. cf. *guizhouense* (slightly cytoplasmic coagulation). To cause cytoplasmic damage to *Pythium* and *Globisporangium*, the protease from *Trichoderma* could play an important role (72). Notably, in the exo-proteomic analysis, proteolysis-related proteins ranged between 4% and 29.71% of the total abundance. With the highest proteolysis-related protein abundance, *T. virens* caused strong cytoplasmic coagulation. Secondary metabolites could also contribute to the antagonism to *Pythium* and *Globisporangium*, as was shown in previous studies (30, 31). However, our experimental set-up focused on the secreted hydrolytic cellulases because we wanted to focus on a target that was unique to oomycetes compared to fungi (oomycetes contain cellulose in their cell walls, whereas true fungal cell walls lack cellulose). Some secondary metabolites are volatile compounds, and we assume in the plate cultures with *Trichoderma* that these volatiles likely diffuse out as the plates were not sealed to the extent we have done in other studies focusing on volatile compounds (73). In the future, it could also be of interest to use the exo-proteomics data set in combination with genome mining to identify protein-based secondary metabolites such as ribosomally synthesized and post-translationally modified peptides (74).

### Conclusion

Antagonism of *Pythium* and *Globisporangium* appears to be a generic property of *Trichoderma* because most of the *Trichoderma* species were at least moderately antagonistic. The study underlines the environmental opportunism throughout the *Trichoderma* genus and suggests that the evolution to acquire cellulases may have contributed to its antagonism and parasitism of *Pythium* and *Globisporangium* species. While the results have uncovered a role for cellulases in the antagonism of *Pythium* and *Globisporangium* species, cellulases did not make a major contribution to *T. reesei* antagonism. Abolishing cellulase activity in stronger antagonists than *T. reesei*, as well as abolishing other antagonism mechanisms that may be compensating for the loss of cellulase activity, should clarify how much cellulases contribute to the antagonism.

## MATERIALS AND METHODS

### Culturing and maintenance of *Trichoderma* and *Pythium* species

*Trichoderma* species, shared from TU Wien collection of industrial microorganisms (TUCIM, Vienna, Austria), were routinely cultured on potato dextrose agar (PDA) at 28°C and maintained as spore glycerol stocks at −80°C for long-term storage. Table 1 lists all 36 *Trichoderma* strains used in this study, along with strain identifiers from public culture collections from where the strains can be obtained. *Pythium* and *Globisporangium* species were routinely cultured on clarified 10% V8 (cV8) juice agar (75) at 25°C and maintained on cV8 agar slants at 12°C. Table 2 lists all three *Pythium* and three *Globisporangium* isolates used in this study, and the isolates are available from the authors on request from our laboratory collection.

### Large-scale confrontation assays of *Trichoderma-Pythium* and *Globisporangium in vitro*

For the preparation of the tested microbes in the confrontation assays, both *Trichoderma* and *Pythium* were pre-cultured on cV8 medium at 25°C for 36 h in the dark. Each 5 mm agar plug covered with hyphae from the hyphal front was transferred onto the opposite side of the 9 cm Petri dish-containing 12.5 mL cV8 medium to initiate the confrontation assays (consisting of 33 *Trichoderma* strains and six isolates of *Pythium* and *Globisporangium* species in 198 combinations of paired dual cultures) and single cultures as controls (*n* = 3). These cultures were incubated at 25°C for up to 15 d under diffuse light (12 h/d). The scoring system was based on the scale of Bell (76). On every odd-numbered day after hyphal contact in the dual culture, an antagonistic score from 1 to 5 was given based on the coverage of the *Trichoderma* colony where 1 is a complete overgrowth of *Pythium* by *Trichoderma*, 2 is a partial overgrowth of *Pythium* and the ⅔ coverage of Petri dish by *Trichoderma*, 3 is a type of deadlock where neither of the species overgrows each other, 4 is a partial overgrowth of *Trichoderma* and the ⅔ coverage of Petri dish by *Pythium*, and 5 is a complete overgrowth of *Trichoderma* by *Pythium*. The *Trichoderma* antagonistic overgrowth was based on the proportion of the *Trichoderma* colony that appeared to be on top of the *Pythium* colony as viewed from the side of the Petri dish with back illumination at this time point. Table S2 contains all the scoring data. The *Pythium* and *Trichoderma* species were hierarchically clustered by using the average antagonistic scores following the “Median” clustering and the “AltGower” distance matrix method at the R pheatmap function on the cloud platform ImageGP (77). The images of dual culture were taken by a Canon EOS R6 camera on 2–4 and 6 d after hyphal contact. Two independent large-scale confrontation assays were done as described above.

### Ecological enrichment analysis of the strongest *Trichoderma* antagonists

Ecological characteristics of each *Trichoderma* strain were categorized by four ecological categories of taxonomic section (sect. *Trichoderma*, sect. Longibrachiatum, sect. *Harzianum Virens*, and sect. Pachybasium) (78), habitat (terrestrial and marine) (61), lifestyle (plant saprotroph, endophyte, and mycoparasite) (79), and distribution (cosmopolitan and limited) (80). Table S3 contains a complete list of the *Trichoderma* spp. and all of their assigned ecological categories.

Using the antagonistic scores given in the large-scale confrontation assay, a *χ*^2^ test calculator (https://www.socscistatistics.com/tests/chisquare2/default2.aspx) was used to identify the ecological sub-categories that were enriched in the strongest *Trichoderma* antagonists at a significance level of *P* < 0.05. The strongest *Trichoderma* antagonists were considered those with an average antagonism score <2.2 toward particular *Pythium* or *Globisporangium* species at the first five time points in the confrontation. The ecological sub-categories of these strongest *Trichoderma* antagonists were compared with the ecological sub-categories of the other *Trichoderma* strains (which had an antagonism score ≥2.2 toward particular *Pythium* or *Globisporangium* species) on each enrichment analysis.

### Pot-trial experiment using *Trichoderma* to control Pythium soft rot of ginger

Ginger seedlings (*Zingiber officinale Roscoe*) of Laiwu big ginger variety obtained from Hubei Academy of Agricultural Sciences were maintained in tissue culture jars at 25°C with light (12 h/d). Note that the description of the method for the pot-trial uses a day numbering system with day 0 corresponding to the transplanting of the seedlings. Each rinsed seedling (the seedlings were ~5 cm in height and with 5–6 green leaves) was transplanted into sterilized wet vermiculite (~100 mL) contained in a 180 mL pot. The pot was placed on an inverted Petri dish lid and was covered with a 270 mL transparent plastic pot to prevent spore cross-contamination. The transplanted seedlings were incubated in a growth chamber at 25°C, with 16 h light (5,000 Lux) per day, and were watered with 5 mg compound fertilizer (Plant-Soul 20–20−20 + TE) dissolved in 5 mL sterilized ultrapure water on days 3 and 6. The air in the growth chamber was continually moistened by water vapor from an open water tank at the bottom of the chamber. To prepare the *T. asperellum* CBS 433.97 inoculum, the strain was cultured on PDA plates at 28°C for up to 7 d under diffuse light (12 h/d), and the spores were harvested using a 0.05% (wt/vol) Tween 20 solution. These harvested spores were diluted using autoclaved tap water to 2.5 × 10^5^ and 2.5 × 10^7^ spore/mL in a total volume of 4 mL and were horizontally frozen at −20°C overnight. On day 7, the frozen *T. asperellum* spore suspensions were slowly allowed to melt into the vermiculite near the base of the stem to give final concentrations in the vermiculite of 1 × 10^4^ and 10^6^ spore/mL. The inoculation of the spores as a slow melting solid prevented the spores from sinking too quickly to the base of the pot. There was no significant loss in germination rate from freezing the spores (data not shown). The frozen autoclaved spore suspension was used as a mock inoculum. To prepare the *P. myriotylum* SWQ7 inoculum, autoclaved wet wheat seeds were added to the mycelial lawn of an actively growing *P. myriotylum* SWQ7 culture and were incubated for a further 7 d at 25°C. On day 14, for the *P. myriotylum* inoculation, four living or autoclaved (inactivated *P. myriotylum* mock control) mycelium-covered wheat seeds were inoculated close to the ginger roots in each pot to a depth of 2–3 cm.

From days 21 to day 28, the above-ground disease symptoms on seedlings were scored based on a modification of the criteria from (81), whereby a score of 1 is the visible chlorosis of the leaf sheath collar and lower leaves, 2 is the visible etiolation of stem and upper leaves, and 3 is the death or total collapsing of the stem. The plant disease index and *Trichoderma* protection rate were subsequently calculated. According to the principles of Koch’s postulates, on day 29, *P. myriotylum* was re-isolated from the roots and rhizosphere of diseased ginger, and *T. asperellum* was re-isolated from the roots and rhizosphere of disease-protected ginger. The roots were surface-sterilized before isolation. The isolated single colonies were used for identification by gDNA extraction and sequencing of the PCR-amplified ITS region. The universal primers ITS1 and ITS4 (82) were used for PCR amplification of the ITS region using the PCR reaction and cycling conditions described previously (83).

### Analysis of the composition of *P. myriotylum* mycelium

*P. myriotylum* SWQ7 mycelium was harvested from at least three shaking liquid cultures after 3 d in cV8 medium, pooled, and rinsed with the autoclaved minimal medium that contained (NH_4_)_2_SO_4_ 5 g/L, KH_2_PO_4_ 15 g/L, MgSO_4_ 0.6 g/L, CaCl_2_ 602.2 mg/L, EDTA 20 mg/L, ZnSO_4_·7H_2_O 8.8 mg/L, MnCl_2_·4H_2_O 2.02 mg/L, FeSO_4_·7H_2_O 2 mg/L, CoCl_2_·6H_2_O 0.64 mg/L, CuSO_4_·5H_2_O 0.63 mg/L, (NH_4_)_6_Mo_7_O_24_·4H_2_O 0.44 mg/L, and was adjusted to pH 5.5 by KH_2_PO_4_ or K_2_HPO_4_. The rinsed mycelium was freeze-dried and ground with a ball mill to a fine powder (diameter <30 µm). This mycelial powder was used as part of autoclaved media for plate and shake-flask cultures described in later sections. The method for preparing the *P. myriotylum* mycelium powder is similar to previous methods for preparing mycelium for inducing gene expression and protein production in *Trichoderma* cultures (84, 85).

To remove soluble components, such as sugars, 50 mg acquired mycelial powder was extracted and sonicated three times with 1.4 mL of 80% (vol/vol) ethanol, then once for 10 min with 1.4 mL of 2:1 chloroform:methanol and once with 1.4 mL of 100% acetone. The centrifugation steps during the extraction were performed for 15 min at 3,000 × *g*. The extracted mycelial powder (1 mg) was hydrolyzed with 1 mL of 4 M trifluoroacetic acid (TFA) for 4 h at 115°C, and the TFA was evaporated, and three washes with 50% methanol were used to remove residual TFA. The re-solubilized sugars were fluorescently derivatized using anthranilic Acid (2-AA) following the procedure of (86) . Monosaccharide detection was performed using a Shimadzu Nexera ultra-high-performance liquid chromatography system equipped with an RF-20Axs fluorescence detector and a reverse-phase C18 HyperClone 5 µm ODS120 Å, 250 × 4.60 mm column (Phenomenex, USA). The mobile phase consisted of Solution A (0.5% phosphoric acid, 1% tetrahydrofuran, and 0.2% n-butylamine) and Solution B (100% acetonitrile) run as a gradient starting from 97.5% Solution A and 2.5% Solution B to 5% Solution A and 95% Solution B, with a flow rate of 1 mL/min. The excitation wavelength was set to 360 nm, with the emission set to 425 nm. The following sugar standards were used: D-arabinose (Ara), D-xylose (Xyl), D-mannose (Man), D-galactose (Gal), D-glucose (Glc), and N-acetylglucosamine (GlcNAc).

### Semi-quantification of *Trichoderma* cellulase activity on various media

0.1% (wt/vol) AZCL-HE-Cellulose (Megazyme, Cat# I-AZCELF), as the cellulase-sensitive dyed substrate similar to its use in a previous study (87), and 0.5% (wt/vol) xanthan gum (Solarbio, Cat# G8800), as the suspending agent, were added to minimal medium or cV8 medium as the AZCL-cellulose media. To investigate the induction of the *Trichoderma* cellulase activity by various substrates, each potential cellulase inducer of 0.4% (wt/vol) non-extracted *P*. *myriotylum* mycelial powder, 1% (wt/vol) cellulose (Sigma, Cat# 435236), 5 mM, and 25 mM of cellobiose (Sigma, Cat# C7252), D-xylose (Sigma, Cat# X1500), lactose (Sigma, Cat# 17814), or the non-inducing D-glucose (Macklin, Cat# D810594), and D-fructose (Macklin, Cat# D809612) was added to the AZCL-cellulose media. For better quantification of the *Trichoderma* cellulase activity in unit area, the square plate with 36 cells (2.5 cm²) was used to contain 15 mL of the various media. To prepare the inoculum used on the AZCL-cellulose plates, the freshly harvested mature spores from the selected *Trichoderma* strains were inoculated into a minimal medium [containing 1% (wt/vol) D-fructose and 0.1% (wt/vol) peptone] and incubated at 200 RPM and 28°C in the dark for up to 20 h. Then 1 × 10^3^ pre-germinated spores were inoculated onto a corner of each AZCL-cellulose plate (*n* = 3) and were incubated at 28°C in the dark for up to 5 d. The images of the *Trichoderma* colonies with the dye-releasing zone were taken by a Canon EOS R6 camera with back illumination from a lightbox.

### Calcofluor white staining and microscopy of confrontations *in situ*

For better hyphal observations *in situ* of dual culture in confrontation assay, the 35 mm microscopic dishes (Nest, Cat# 706001) were used in this study for microscopic investigation. Each 3 mm agar plug covered with hyphae from the hyphal front was transferred onto the opposite side of the 35 mm dishes containing 1 mL cV8 agar medium to initiate the confrontation assays (consisting of 7 selected *Trichoderma* strains and the selected host *P. myriotylum* SWQ7 as paired dual culture) and single cultures as control (*n* = 5). These cultures were incubated at 25°C for up to 7 d under diffuse light (12 h/d). The images of cultures were taken under a Nexcope NE900 microscope. For clear morphological discrimination of two partners in the dual cultures, mycelia were stained with 300 µL calcofluor white stain (0.1% calcofluor white MR2 and 0.05% Evans blue; MKbio Cat# MM1011), based on method from (88) and modified mainly by the addition of Evans blue, and 300 µL 10% (wt/vol) NaOH for 1 min, and gently rinsed by sterile 0.9% (wt/vol) NaCl (0.9% normal saline), and were observed and recorded under a Nikon E400 microscope with a DAPI filter. *P. myriotylum* hyphae can be distinguished from the *Trichoderma* hyphae by its oospore structures and the absence of septa in its hyphae.

### Shake-flask cultures containing autoclaved *P. myriotylum* mycelia for induction of *Trichoderma* enzymes

Freshly harvested mature spores from selected *Trichoderma* strains (1 × 10^4^ spores/mL) were used to inoculate pre-cultures in 50 mL minimal medium containing 1% (wt/vol) D-fructose and 0.1% (wt/vol) peptone in a 250 mL flask. The pre-cultures were incubated at 200 RPM and 28°C in the dark for 48 h except for *T. parepimyces*, which was incubated for 96 h pre-culture. The mycelia were filtered through a nylon net and were rinsed with a minimal medium. One gram wet weight of *Trichoderma* mycelia (corresponding to ~0.06 g dry weight) was inoculated into 50 mL autoclaved minimal medium containing 0.4% (wt/vol) non-extracted *P*. *myriotylum* mycelial powder as the inducer in a 250 mL flask and was incubated at 28°C and 200 RPM for 48 h (*n* = 3). The two-step culturing method (i.e., a pre-culture and then a culture with the inducing substrate) is similar to a previous method using *Trichoderma* and an inactivated mycelium-based inducer (89). For further investigation of the inductive enzymes from *Trichoderma*, the supernatant was harvested from liquid cultures at 48 h by centrifugation at 4°C and 7,200 × *g* for 10 min and was flash frozen in liquid nitrogen. Later, 36 mL of the supernatant from each inductive culture was precipitated by 20% (vol/vol) trichloroacetic acid solution (Sigma, Cat# T0699) and centrifugation at 4°C and 7,200 × *g* for 10 min. Then, the pellet was extracted with 1 mL of cold acetone twice with centrifugation at 4°C and 20,000 × *g* for 10 min and finally resuspended in 100 µL of 8 M urea.

### *Trichoderma* exo-proteomic analysis

For visual inspection of inductive liquid cultures inoculated with selected *Trichoderma* strains, an equal volume of urea re-solubilized exo-protein samples with the 5× protein-loading buffer (with dithiothreitol; Coolaber, Cat# SL1170) was loaded on the 10% SDS-PAGE gel (Zomanbio, Cat# ZD304C-12) and was run in the 1× SDS-PAGE running buffer (SDS 0.1%, Tris 25 mM, Glycine 0.25 M, pH = 8.6) under denaturing conditions, alongside with the positive control (0.25 µg bovine serum albumin) and a protein marker (11–180 kDa; Solarbio, Cat# PR1910). Then, the various banding patterns on SDS-PAGE gel were stained by using a silver staining kit (Beyotime Cat# P0017S).

For further exo-proteomic analysis, urea re-solubilized exo-proteins samples were analyzed by the Applied Protein Technology Company (China) using label-free LC-MS/MS. The samples were digested by trypsin and separated using an Easy nLC (Thermo Fisher Scientific) for 120 min and identified using a Q Exactive mass spectrometer. The peptides were loaded onto a reverse phase trap column (Thermo Scientific Acclaim PepMap100, 100 µm × 2 cm, nanoViper C18) connected to the C18-reversed-phase analytical column (Thermo Scientific Easy Column, 10 cm long, 75 µm inner diameter, and 3 µm resin) in 0.1% formic acid and separated with a linear gradient of 84% acetonitrile and 0.1% formic acid at a flow rate of 300 nL/min, controlled by IntelliFlow technology. Data were acquired using a data-dependent top-10 method dynamically choosing the most abundant precursor ions from the survey scan (300–1,800 m/z) for HCD fragmentation. The automatic gain control target was set to 3 × 10^6^, and a maximum inject time was set to 10 ms. Dynamic exclusion duration was set to 40.0 s. Survey scans were acquired at a resolution set at 70,000 at m/z 200, and the resolution for HCD spectra was set at 17,500 at m/z 200, and the isolation width was 2 m/z. The normalized collision energy was 30 eV, and the underfill ratio, which specifies the minimum percentage of the target value likely to be reached at maximum fill time, was defined as 0.1%. The instrument was run with peptide recognition mode enabled.

Identification and quantitation of proteins for each sample were combined and searched using the MaxQuant 1.5.3.17 software (90). The GeneCatalog protein sequences from the JGI MycoCosm database were used as Trias1 (57) for *T. asperellum* CBS 433.97, Triat2 (56) for *T. atroviride* IMI 206040, Trigui1 (57) for *T*. cf. *guizhouense* NJAU 4742, Tripare1 for *T. parepimyces* CBS 122769, Trire2 (91) for *T. reesei* QM6a, and TriviGv29_8_2 (56) for *T. virens* Gv29-8. GO terms were annotated with Blast2GO (https://www.blast2go.com) (92). The protein domains were predicted using Interproscan (93). Subcellular localization was predicted by CELLO software (94). The proportion of major antagonism-related GO functional categories in the exo-proteins was expressed as a percentage of the total label-free quantification intensity values.

### *Trichoderma* ortholog analysis

For ortholog analysis of GeneCatalog proteins sequence, JGI MycoCosm fasta data for selected *Trichoderma* species were loaded to the software OrthoFinder (2.5.4) (95) using the Galaxy web platform (https://usegalaxy.eu) (96). The inflation parameter in the software was set to 1.5. Three categories of orthogroups were defined: the core ones (shared in all selected *Trichoderma* species), the dispensable ones (found in at least two to five selected species), and the unique ones (only found in one selected species), and the results were listed in Table S1. The exo-proteins were ranked by abundance for each strain and labeled by orthogroups to demonstrate changes in the relative abundance of orthologous exo-proteins.

### Culturing and analysis using *T. reesei* Δ*xyr1* mutant

The *T. reesei* Rut-C30 (carbon catabolite derepressed *cre1* mutant), its *xyr1* gene-deletion mutant, and the complemented mutant were gifted from Ma et al. (54). The same large-scale confrontation assay toward six tested *Pythium* and *Globisporangium* isolates described in a previous section was performed with the *xyr1* mutant and control strains on cV8 plates and cV8 plates supplemented with 5 mM of D-xylose.

### Statistical significance analysis

Statistical significance of the data within bar charts and line charts was evaluated by analysis of the variance with Tukey’s post hoc test with the level of significance fixed at 0.05. The same letter label indicates no significant difference among data points.

## Data Availability

The raw mass spectrometry data set for exo-proteomics has been deposited in the PRoteomics IDEntification Database (PRIDE) as project PXD051895.

## SUPPLEMENTAL MATERIAL

Supplemental material aem.00681-24-s0001.pdf Captions for Tables S1 to S4; Figures S1 to S4.

Table S1 aem.00681-24-s0002.xlsx Ortholog analysis.

Table S2 aem.00681-24-s0003.xlsx Raw antagonism scores.

Table S3 aem.00681-24-s0004.xlsx Ecological enrichment analysis.

Table S4 aem.00681-24-s0005.xlsx Exo-proteomics dataset.

ASM does not own the copyrights to Supplemental Material that may be linked to, or accessed through, an article. The authors have granted ASM a non-exclusive, world-wide license to publish the Supplemental Material files. Please contact the corresponding author directly for reuse.
