# Supplementary material for: Genus-wide analysis of Trichoderma antagonism toward Pythium and Globisporangium plant pathogens and the contribution of cellulases to the antagonism
Source: Appl Environ Microbiol. 2024 Aug 7;90(9):e00681-24. doi: 10.1128/aem.00681-24 (PMC11409678; doi:10.1128/aem.00681-24)
Supplement: Supplemental material — Captions for Tables S1 to S4; Figures S1 to S4. [file aem.00681-24-s0001.pdf]

Table S1. (Separate Excel file) List of Orthofinder analysis with the grouping of the orthologous protein from six selected *Trichoderma* species *T. asperellum*, *T. atroviride*, *T. guizhouense*, *T. parepimyces*, *T. reesei*, and *T. virens* (Excel file).

Table S2. (Separate Excel file) The raw data from the scoring of the antagonism between *Trichoderma* and *Pythium* or *Globisporagium* species (Excel file). The following Excel nested IF function was used to categorize the *Trichoderma* colony coverage for Bell's scale where LoT = the length of *Trichoderma* colony coverage: '=IF(LoT>8.1,1,IF(LoT>5.9,2,IF(LoT>4.4,2.5,IF(LoT>2.9,3,IF(LoT>0.5,4,5))))).

Table S3. (Separate Excel file) Summary of the ecological categorization of the *Trichoderma* species listed alongside the average antagonistic score towards each *Pythium* or *Globisporagium* isolate. The sheet containing the data from the confrontation between *Trichoderma* species and *P. myriotylum* SWQ7 also contains citations to literature that support the ecological categorization of the species. Enrichment for Trichoderma species with a cosmopolitan distribution among the strongest antagonists. Ranking of the average antagonistic scores evaluated for the *Trichoderma* strains in the dual culture with each *Pythium* or *Globisporagium* isolate. P value of the enrichment scores: ***, $P < 0.001$; **, $P < 0.01$; *, $P < 0.05$. Note: "sect. Pachybasium" excludes those in "Harzianum Virens". The white boxes on the ecological categorization bars represent where conclusive information was not available. The antagonistic scores were the average from two independent experiments, and there was a significant positive correlation ($R^2 = 0.5412$, $P < 0.0001$, n = 198) between the antagonistic scores from the first and second experiments (Fig. S1) (Excel file).

Table S4. (Separate Excel file) Summary of exo-proteomics data for the *Trichoderma* exo-proteins sample extracted from 48 h liquid culture induced with 0.4% w/v *P. myriotylum* mycelial powder. The exo-proteins from three biological replicate shake-flask cultures were analyzed (Excel file).

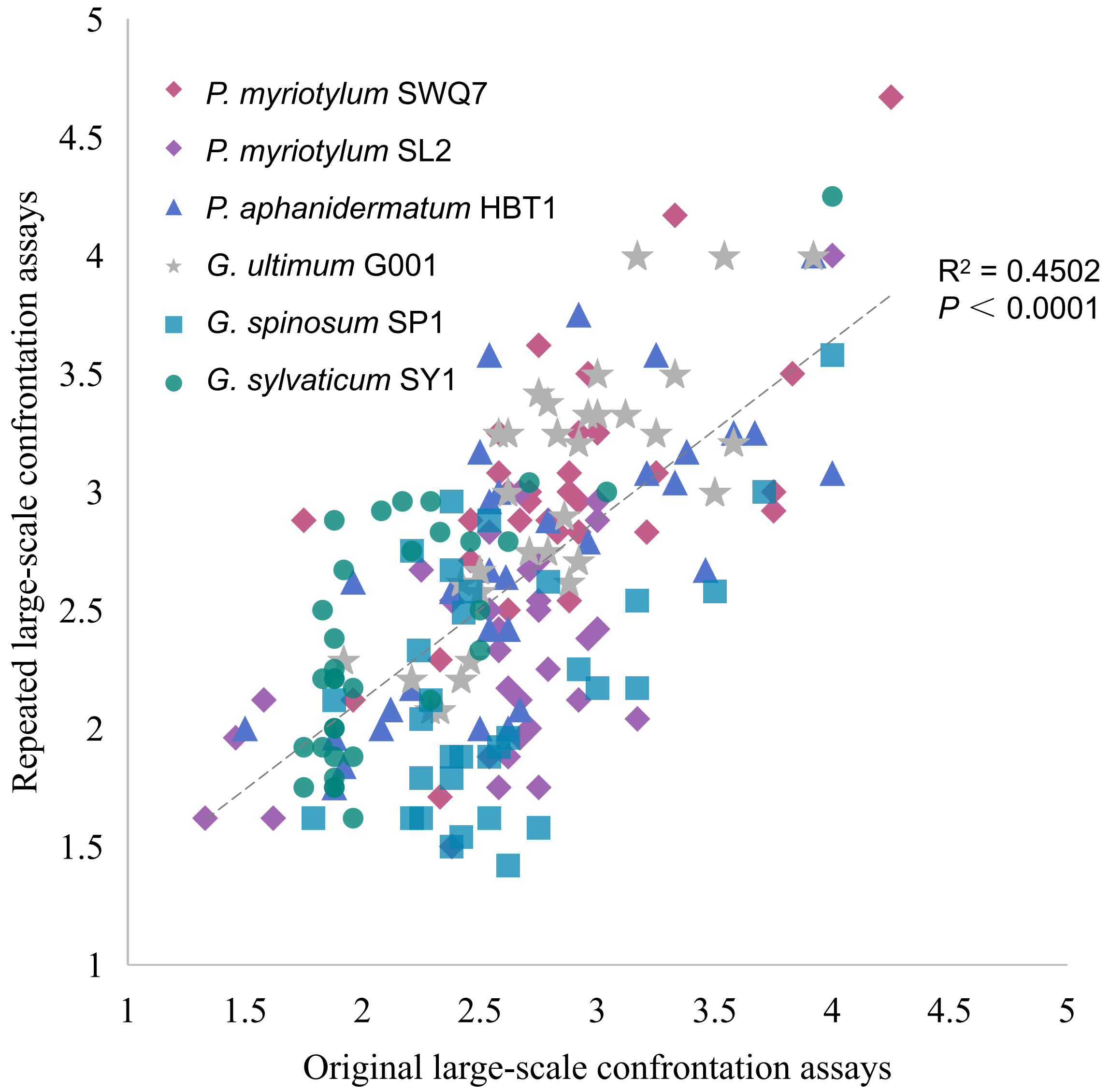

Fig. S1. Scatter plot and correlation of the average antagonistic scores from the original (x-axis) and repeated (y-axis) large-scale confrontation assays between *Pythium or Globisporangium* and *Trichoderma*.

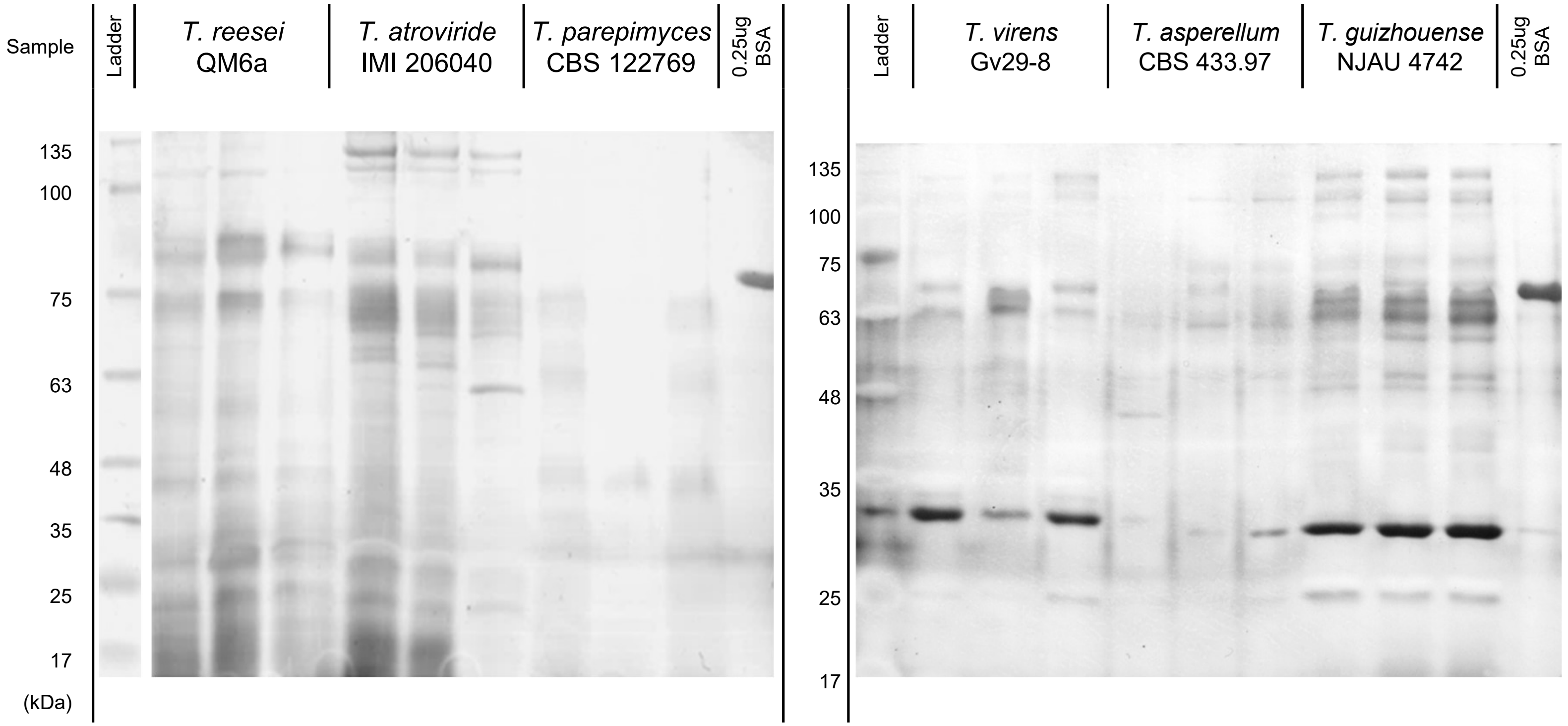

Fig. S2. SDS-PAGE gel after silver staining of the urea-resolubilized precipitated *Trichoderma* exo-protein samples used in exo-proteomics. The three adjacent lanes of each species on the PAGE gel are from individual biological replicate flasks. An equal volume of the urea-resolubilized precipitated proteins was loaded in each well which can be considered representative of the protein concentration in the shake flasks because an equal volume of culture filtrate was originally precipitated from each of the cultures.

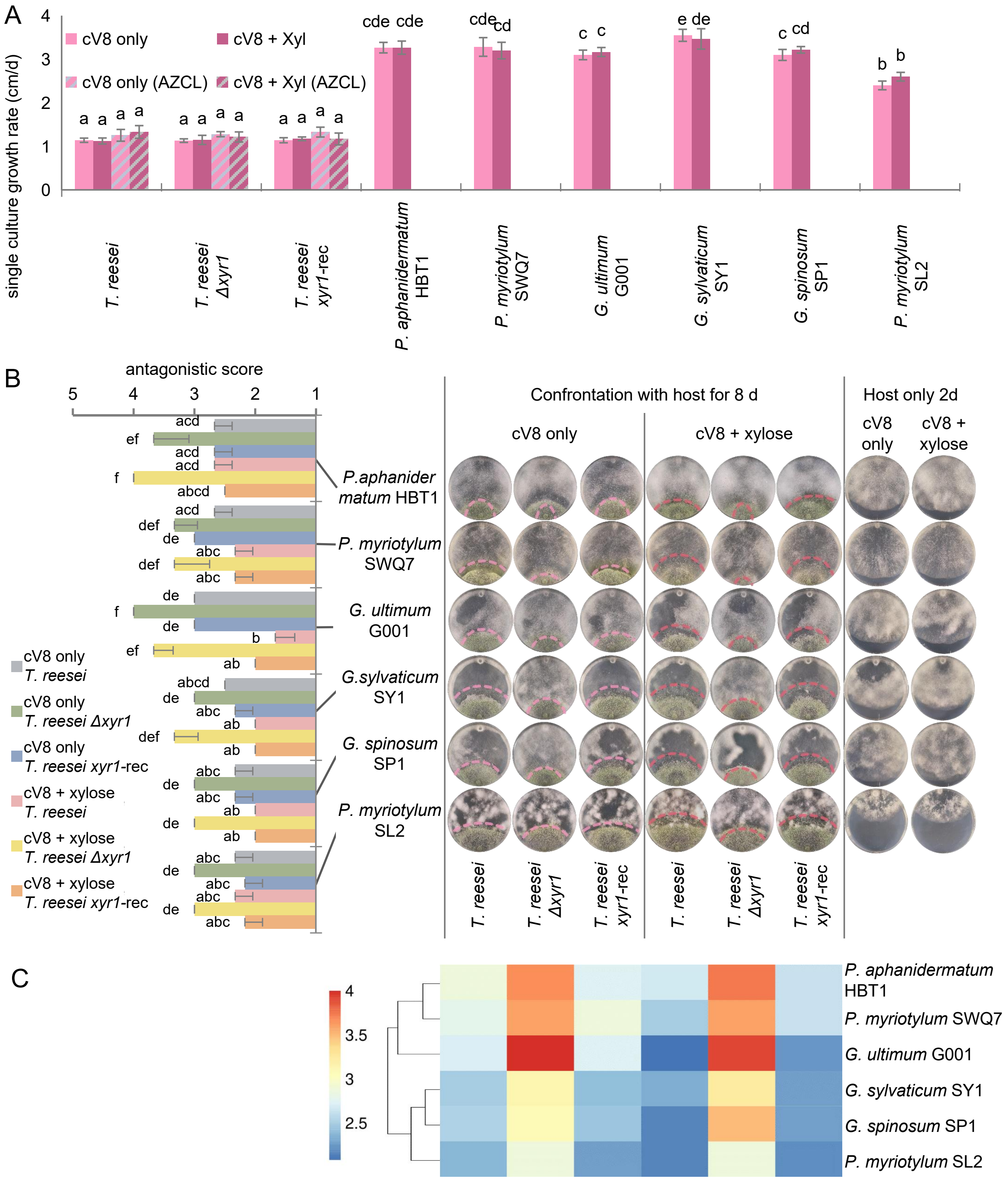

Fig. S3. The whole confrontation assay between *T. reesei* RUT-C30 Δ*xyr1* mutant with its controls and six *Pythium* and *Globisporangium* isolates. (A) Single culture growth rate (cm/d) of the tested *T. reesei*, *Pythium* and *Globisporangium* colonies, and error bars represent standard error from three replicate plates. (B) Plate culture images of *T. reesei* mutants and *Pythium* or *Globisporangium* species on cV8 only and supplemented with 5 mM D-xylose, alongside the antagonistic score represented by the bar chart. Dual culture images were recorded 3 d after the colonial contact. The confrontation front was labeled with a dotted line. The plate images are representative of three plates. (C) Heatmap of hierarchical clustering by the pattern of average antagonistic score evaluated for the *Trichoderma* mutants in the confrontation assays, clustering by *Pythium* and *Globisporangium* in columns (cluster method: median, distance matrix method: algower). Statistical significance was evaluated by ANOVA with Tukey's post hoc test with the level of significance fixed at 0.05. xyr1-rec = *xyr1* recomplemented mutant.

A

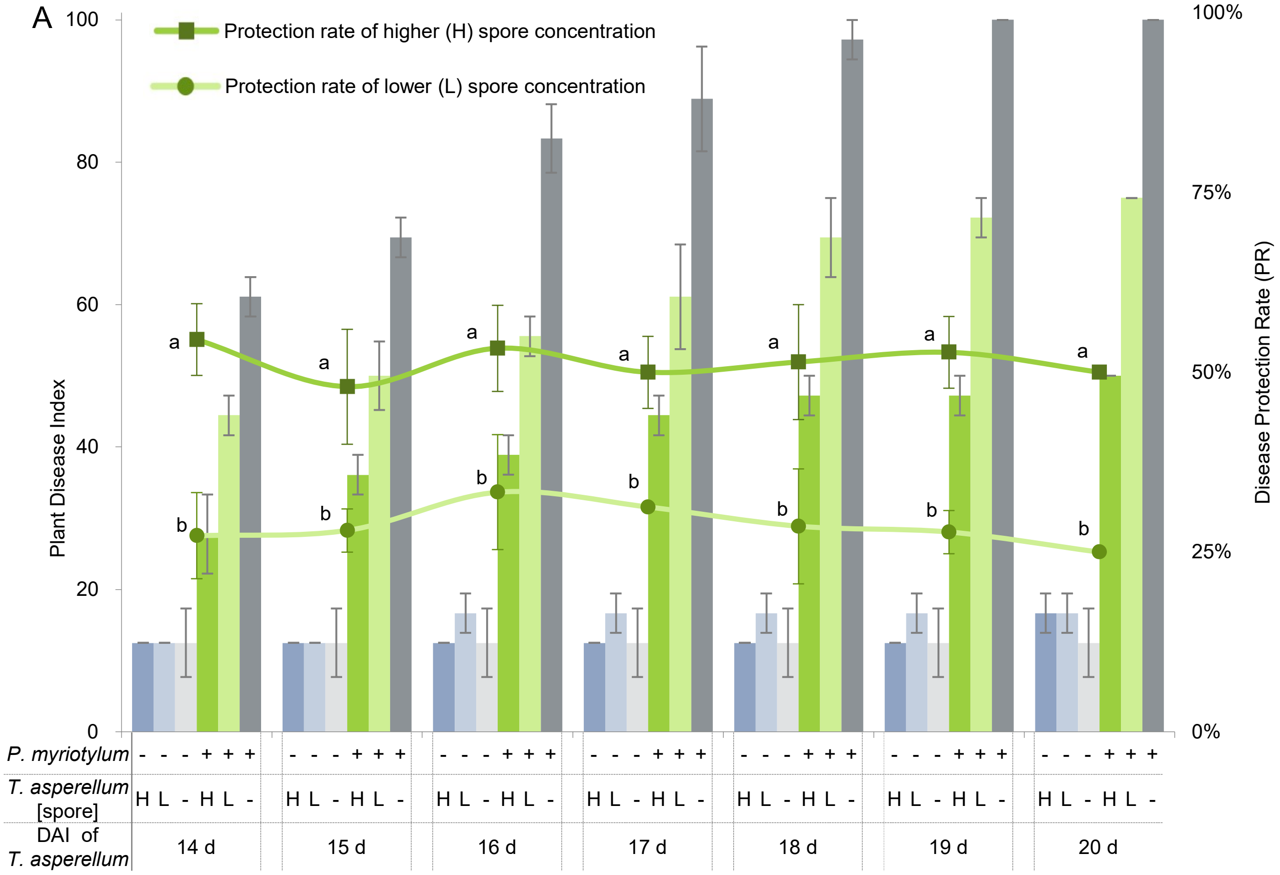

B

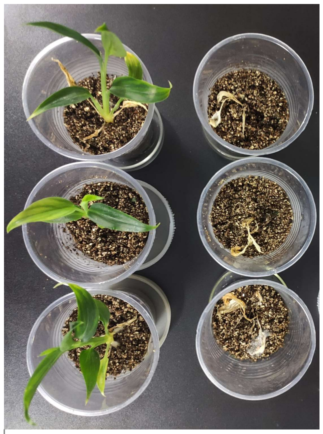

P. myriotylum	+	+
T. asperellum (H = higher [spore])	H	-

Fig. S4. The pot-trial experiment using *T. asperellum* CBS 433.97 to control ginger soft rot caused by *P. myriotylum* at 14 to 21 d after the inoculation of *T. asperellum* spores. (A) The Disease Index (≤100, represented by the bar chart, where error bars represent the standard error, n = 3) from the scoring of the disease symptoms, followed by *T. asperellum* Disease Protection Rate of ginger (PR ≤ 100%, represented by the line chart, and its error bars represent the standard error). Statistical significance was evaluated by ANOVA with Tukey's post hoc test with the level of significance fixed at 0.05, n = 3. (B) Representative images (21 d) of 3 of the 12 replicate plants inoculated with *P. myriotylum* and *T. asperellum* or *P. myriotylum* only (control). Twelve replicate plants were used for each treatment, and the calculation of the Disease Index and Protection Rates, these replicates were grouped into three groups (n= 3) containing four plants. Higher (H = $1x10^6$ spore/mL) and lower (L = $1x10^4$ spore/mL) *T. asperellum* spore concentrations were inoculated, and the concentration is within the vermiculite. This experiment was repeated twice with similar results each time.
